# The SpikerBox: A Low Cost, Open-Source BioAmplifier for Increasing Public Participation in Neuroscience Inquiry

**DOI:** 10.1371/journal.pone.0030837

**Published:** 2012-03-21

**Authors:** Timothy C. Marzullo, Gregory J. Gage

**Affiliations:** Backyard Brains, Inc., Ann Arbor, Michigan, United States of America; Dalhousie University, Canada

## Abstract

Although people are generally interested in how the brain functions, neuroscience education for the public is hampered by a lack of low cost and engaging teaching materials. To address this, we developed an open-source tool, the SpikerBox, which is appropriate for use in middle/high school educational programs and by amateurs. This device can be used in easy experiments in which students insert sewing pins into the leg of a cockroach, or other invertebrate, to amplify and listen to the electrical activity of neurons. With the cockroach leg preparation, students can hear and see (using a smartphone oscilloscope app we have developed) the dramatic changes in activity caused by touching the mechanosensitive barbs. Students can also experiment with other manipulations such as temperature, drugs, and microstimulation that affect the neural activity. We include teaching guides and other resources in the supplemental materials. These hands-on lessons with the SpikerBox have proven to be effective in teaching basic neuroscience.

## Introduction

**Figure 1:**
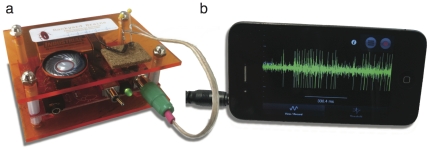
The SpikerBox. Depiction of the SpikerBox (a) and iPhone running custom open-source iOS software (b) used for electrophysiology experiments in the classroom.

Not only is neuroscience absent from most K-12 curricula, but even in college, students must wait until upper level science courses to gain exposure to principles of brain function [1]. We hypothesized that neuroscience education was missing from K-12 curricula not because of a lack of interest [2], but due to a lack of simple, compelling, and inexpensive tools to investigate and understand neurons. Entry-level neurophysiology equipment used for teaching neuroscience typically costs >$3,000 and requires significant training to use and understand. While students can observe the anatomical brain structure from models or preserved tissues, students cannot observe the dynamic electrical structure of the brain without conducting experiments on living neurons firing action potentials. This presents a challenge for K-12 teachers as regulatory restrictions prevent using standard vertebrate models (rats, mice, fish, and frogs) in the classroom for *in vivo* experiments. In order to overcome these issues, we developed a low-cost tool, the SpikerBox (Figure 1), that enables students and teachers to easily listen to and record invertebrate action potentials (“spikes”) with minimal dissection skill and training.

A current reality in the global economy of the 21st century is a dearth of students successfully completing post-secondary degrees in STEM-fields (science, technology, engineering, and mathematics) in the United States [3]. In response, President Obama's administration created new programs to boost STEM-related education and tools as part of the “Educate to Innovate” campaign [4], [5]. Neuroscience is uniquely positioned to serve as a “model discipline” for engaging students, subsequently improving performance in STEM-related fields, by combining biology, physics, electronics, health, and mathematical modeling in a single compelling field.

A widespread effort is growing to bring neuroscience into the public arena [6], [7], [8], [9]. For example, the Society for Neuroscience and The Dana Alliance for Brain Initiatives have partnered to bring Brain Awareness Week every year to communities around the country, which offers opportunities for teachers and students to participate in engaging, hands-on neuroscience educational activities with scientists [10], [11]. Recently, universities have partnered with high schools to run interactive K12 neuroscience workshops [7], [12]. The Society for Neuroscience has also created a “virtual encycloportal” [13] that in one location catalogs a diverse array of resources to assist teachers in the development of neuroscience lesson plans. Such efforts can successfully give some students a brief exposure to neuroscience, but equipment to bring neuroscience education to all students is still lacking.

We were motivated to develop the SpikerBox not only as a low-cost tool to study neuroscience, but also because hands-on activities enhance student learning [14], [15], [16], [17]. As such, this manuscript is designed in two parts. The first describes experiments that employ the SpikerBox in a secondary classroom setting. We discuss our observations about the utility of our devices and provide survey feedback on learning outcomes. The second part, included as appendices, provides the experimental protocols (adapted from our experiment wiki [18]) for students to use as a guide.

## Materials and Methods

### 1. Participants

The classroom workshops, typically lasting 3–6 hours, were coordinated and run with local high school teachers. Our workshops comprised of interspersed lectures on neural engineering (how to experiment on and interface with neurons), simple electronics (how transistors, resistors, and capacitors function), and basic neuroscience (the structure and function of neurons, focusing on electrophysiology). Between these brief 15–20 minute lectures, students assembled their own SpikerBoxes, which they were allowed to keep. While students assembled their SpikerBoxes, we along with the teacher taught principles of soldering and electronics assembly (avoiding shorts, how to stay electrically grounded, etc…). Upon completion and testing of their assembled SpikerBoxes, students then worked together in small groups to perform experiments. Survey data were collected from students in two high school lectures. All educational research testing was done under informed consent of the high school students and teachers, and our policies have been reviewed and approved by the Albion College IRB Review Board. The demographics of the two schools districts were [96% White, 2% Hispanic, 2% Other] and [87% White, 3% Black, 5% Hispanic, 2% Asian, 3% Other] [19].

### 2. Equipment

For electrophysiological measurements, we used an open-source bioamplifier “SpikerBox” which we developed in house (Figure 1a). Complete parts list and schematics are available in this document for self-assembly (File S1 – Circuit Diagram and Guide, Files S6, S7, S8, S9 – CadSoft Eagle files and parts list for printing circuit boards), or can be obtained commercially through Backyard Brains. The three-stage amplifier is band-pass filtered between 300–1300 Hz and contains a speaker to make the neural activity audible. An output port is available for laptop or smartphone recording with ∼900× amplification. Neural data for these experiments were recorded using an iPhone (Figure 1b) and the open-source Backyard Brains app [20]. An iPod Touch, iPad, or Android device can also be used [21]. The electrodes interfacing with the invertebrate preparations consist of two stainless steel standard sewing needles (0.6 mm diameter) or small #15 beading needles (0.25 mm diameter) soldered to a standard 24-gauge stranded speaker wire. The electrodes have a typical DC resistance of 0.3 Ω and a 1 kHz impedance of 20–30 kΩ. For microstimulation, a standard MP3 player (iPod/iPhone/Android) or a laptop can be used to deliver stimuli.

### 3. Experiments

While our experiment inventory continues to grow [18], we focus here on the four pedagogical activities selected to highlight basic neuroscience concepts. The background, illustrations, procedural information, and some student questions for these experiments are included in files S2, S3, S4, S5.

#### 3.a. Experiment I

Guiding Question: How do nerves carry information about touch?

Learning Goals: Students will be able to: 1) understand how sensations enter into the nervous system, and 2) describe the concept of rate coding.

Activity: Students learn the concept of “rate-coding” by listening to the changes in neural discharge in response to pressure of touch using the cockroach leg preparation. Location-dependent (or “somatotopic”) responses can be illuminated by touching several areas along the leg.

#### 3.b. Experiment II

Guiding Question: How do neurons generate electricity?

Learning Goals: Students will be able to: 1) explain the way that neurons produce action potentials through ion channels, and 2) describe why low temperatures *temporarily* prevent a neuron from producing action potentials, while high temperatures *permanently* prevent a neuron from producing action potentials.

Activity: By selectively heating and cooling the cockroach leg, students can indirectly observe the properties of ion channels and their influence on spikes.

#### 3.c. Experiment III

Guiding Question: How does your brain tell your muscles to move?

Learning Goals: Students will be able to: 1) describe how electrical stimulation can cause neural activation and muscle contraction, and 2) design an experiment testing ideal frequencies for stimulation.

Activity: By using the analog output of a portable MP3 device or laptop, students can measure the effect of frequency and amplitude on the initiation of movements in the cockroach leg. The use of electricity to create neural or muscle activity is called “microstimulation”.

#### 3.d. Experiment IV

Guiding Question: How do drugs affect neurons?

Learning Goals: Students will be able to 1) describe how neurotransmitters affect synapses, 2) explain how the differential expression of synaptic receptors can alter neuronal responsiveness to compounds, and 3) design an experiment testing their own hypothesis on how a substance may affect the nervous system.

Activity: The effects of “neuroactive” agents on the central nervous system can be studied to explain the function of synapses and neurotransmitters. Two types of neuroactive agents, nicotine and glutamate, are injected into the CNS of a cricket. Students can analyze the behavioral and neural recording results to determine the effects of each.

### 4. Survey Methodology

Students were given ten minutes to fill out a short answer survey at the beginning of the workshop. In one class, an additional survey was administered at the end of lecture/workshop. The pre- and post-surveys consisted of three open ended questions covering one general question about electronics and two on neuroscience. The answers were scored (blinded to pre or post) as ‘0 = incorrect’, ‘0.5 = partially correct’ and ‘1 = correct’ (Table 1). A “knowledge score” was determined as the sum of correct answers and ranged from 0 to 3. An independent samples t-test was conducted to compare knowledge scores before and after the demonstration. An additional (ungraded) question in the post-survey asked “What additional experiment would you like us to design?”

**Table 1:** Grading rubric for the pre- and post-test questions.

Question	Correct (1 point)	Partial (0.5 points)	Incorrect (0 points)
1) How do neurons encode information?	mentioned both chemical neurotransmission and electrical transmission	mentioned either chemical or electrical transmission	mentioned neither chemical nor electrical transmission
2) What are the roles of synapses?	transfer/modify information between neurons with neurotransmitters	mentioned either the space between neurons or neurotransmitters	mentioned neither the space between neurons nor neurotransmitters
3) What is a transistor and how does it work?	mentioned both semiconductors and amplification	mentioned either semiconductors or amplification	mentioned neither semiconductors nor amplification

The answers to the three questions we asked students regarding neuroscience and electronics.

## Results

During our workshops, we found that approximately 75–85% of attendees could build their SpikerBox correctly from scratch without any errors. The remaining 15–25% had minor failures such as incorrectly placed chips or solder shorts. We expected this, since for many students this was their first experience with building electronics and soldering. Through visual inspection, together with the teacher, we easily identified errors and made fixes using an inexpensive desoldering tool (*e.g.* Radio Shack #64-2098). These mistakes were then used as teaching tools to explain how electronics work and why the failure occurred. By the close of the workshops, every student had a working SpikerBox.

### 1. Experimental Results

Four species of cockroaches were used in our experiments: *Americana periplaneta* (American cockroach), *Blaberus discoidalis* (discoid, or false death's head), *Blaberus fusca* (dwarf cave roach) and *Gromphadorhina portentosa* (Madagascar hissing cockroach). The experiments in this study were predominantly performed on *Blaberus discoidalis*, as this species is large, easy for students to handle, easy to maintain, and available from suppliers (such as aaronpauling.com) as a feeder insect. For the neuropharmacology experiment, we used the commonly available house cricket *Acheta domesticus*.

**Figure 2:**
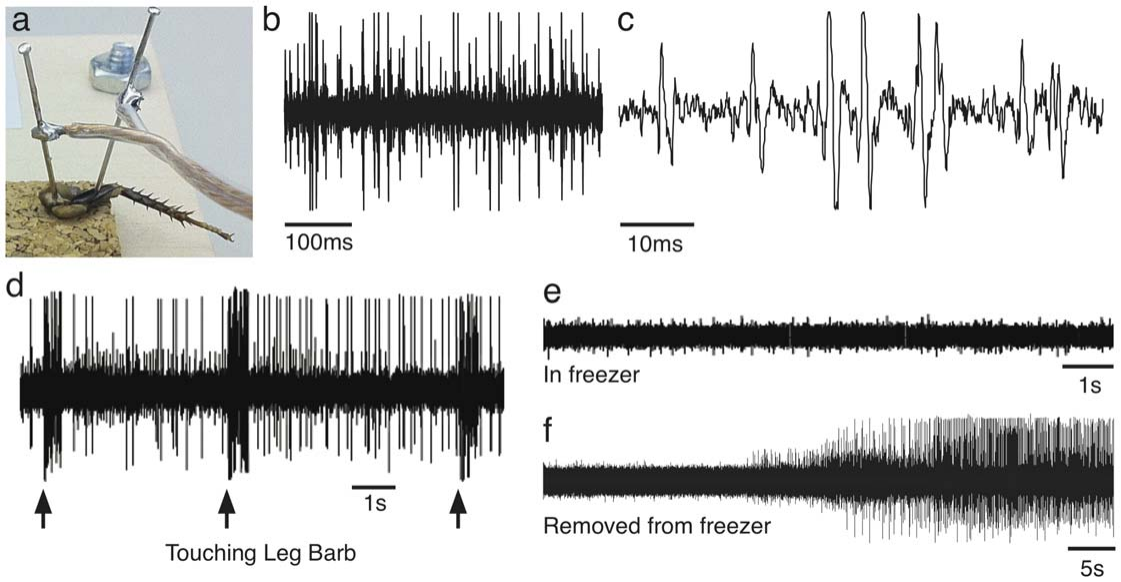
Electophysiological Recordings. (a) Placement of needle electrodes in the cockroach leg prep. (b) Spontaneous firing of action potentials (c) Same data as b, but zoomed in to reveal individual spikes. (d) Neural activity driven by touching mechanosensitive barbs. Arrows indicate when time of touch occurred. (e–f) Neural activity recorded inside of freezer (e) shows no activity, but recovers (f) after returning to room temperature. Data recorded from iPhone and are in arbitrary A–D units.

Preparations for all experiments using a cockroach leg (Experiments I–III) began with students anesthetizing the cockroach in ice water, which led to a discussion on the ethics of animal testing. Students hypothesized various reasons for placing the cockroach on ice, which generated a lively discussion about the use of anesthesia in hospitals and research laboratories. Once the cockroach was anesthetized, a student used scissors to remove one of the hind legs. The cockroach was then returned to its home cage; we explained that the cockroach could still live and reproduce normally when missing 1–2 legs. The SpikerBox electrodes were placed in two positions (Figure 2a) in the detached cockroach leg. When the SpikerBox was switched on, students observed spontaneous neural activity either through hearing spikes via the built-in speaker or seeing spikes displayed on a laptop or a mobile device (Figures 1b, 2b–c).

#### 1.a. Experiment I - How do neurons carry information about touch?

By gently blowing on the leg, or touching the mechanosensitive (touch sensitive) barbs of the leg with a toothpick, students evoked neural activity (Figure 2d). The rate coding model of neuronal firing communication states that as the intensity of a stimulus increases, the frequency (rate) of action potentials, or “spike firing”, increases. Students observed this as they varied the amount of pressure on the barbs.

Somatotopic arrangement is the maintenance of spatial organization within the central nervous system (CNS). Students monitored the neural inputs *en route* to the CNS via the SpikerBox, and determined that the response to touching the mechanosensitive barbs was spatially dependent. Typically, 1–2 barbs of the ∼30 barbs on the tibia were the most sensitive to manipulation (Figure 2d).

#### 1.b. Experiment II - How do neurons generate electricity?

Students selectively heated and cooled the cockroach leg and observed the effect of temperature on spike rate. Typical baseline firing slowed as temperature was lowered from room temperature until it disappeared entirely at ∼20°F (Figure 2e). The mechanism for this decrease in firing can be attributed to the slowing of the ion channel kinetics used to generate action potentials. It should be noted that a cooler of ice (∼32°F) was not sufficiently cold enough to stop firing, therefore students used either an actively powered freezer or a cooler of dry ice (CO_2_).

Bringing the leg back up to room temperature restored the basal rate (Figure 2f). Heating the leg up to ∼100°F increased background firing activity (using a candle placed ∼6 inches below the leg), and heating above 120°F caused neural death. Often, students would hear a dramatic (and characteristic) “scream” of neurons as they massively discharged. This can be explained as the high temperature causing the ion channels and cell membranes to break down.

#### 1.c. Experiment III - How does your brain tell your muscles to move?

In this module, students measured the effect of frequency and amplitude on the electrical stimulation of excitable tissue. By using a simple breakout cable to connect the analog output of an iPod or laptop to the electrode pins in the cockroach leg, current can flow across the pins to generate a voltage that stimulates the tissue. As an opening demonstration, we played hip-hop music from the iPod. The electric signal travelled from the iPod through the electrodes, which caused the detached leg to “dance” (in beat) to the low frequencies of the bass rhythm. We demonstrated that music lacking bass frequencies (e.g. Baroque classical music) was not as effective in eliciting movement. The compelling demo ignited enthusiastic curiosity in the students.

Students then carefully mapped the frequencies/amplitudes that caused evoked movements by playing small tone bursts. The results indicated that the lower frequencies (20–200 Hz) needed a smaller amplitude (less current) to cause a muscle twitch, while higher frequencies (>5 kHz) had no effect at even the largest amplitude. This difference could be explained by the wavelength of the current's cathodic (positive) and anodic (negative) phases. The voltage accumulated inside the excitable tissue is dependent on how long the current was in one phase (cathodic vs. anodic). During low frequencies, the current change moves slowly enough to cause the neural and muscle extracellular voltages to reach threshold. At high frequencies, the currents move too quickly for the extracellular voltage to reach threshold.

#### 1.d. Experiment IV - How do drugs affect neurons?

**Figure 3:**
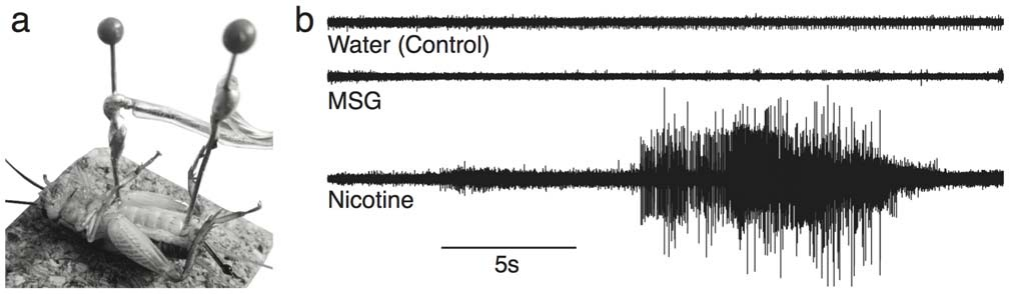
Neuropharmacology Experiment. (a) Placement of recording electrodes in the cricket preparation. (b) Sample traces with control (tap water), saturated monosodium glutamate (MSG) solution, and the nicotine solution injected into a cricket. Injection occurred at t = 0 (beginning of trace). Injections occurred sequentially in the same cricket, beginning with the control, then the MSG, and followed by the nicotine. Injections were approximately 1–2 minutes apart.

For this module, we found that the cricket cercal system was the easiest preparation for the students to test the differential effects of neuroactive agents. Crickets were anesthetized similarly to the cockroach. Figure 3a illustrates the electrode placement and neural recordings in the cricket. The electrodes were placed in the middle of the thorax and the posterior end of the abdomen along the central axis of the insect. Students could then hear neural responses by blowing on the cerci (the long hairs on the posterior end of the cricket).

The students injected solutions that bind to common neurotransmitter receptors, and noted the neural response. The first experiment was with a saturated monosodium glutamate (MSG) solution; excess glutamate ions could then bind to glutamatergic receptors in synapses. The second solution contained a tobacco cigarette (containing nicotine) broken up and soaked in water for 1 d. Nicotine is an agonist that binds to nicotinic acetylcholine receptors.

Because the excitatory neurotransmitter in the *vertebrate CNS is* glutamate, the students had expected the neural firing to increase dramatically when glutamate was injected (Figure 3b). However, glutamate had no effect. It was the nicotine solution that caused a massive excitotoxic discharge. The reason for this perplexing outcome is that the excitatory CNS neurotransmitter of insects is acetylcholine, not glutamate [22]. We explained that nicotine is produced by plants to act as a natural insecticide to defend against insects.

### 2. Survey Results

We assessed the effectiveness of our materials by comparing the knowledge scores (total points) that were collected both before and after the demonstration. There was a significant difference in the pre-test (M = 1.1, SD = .74) and the post-test knowledge (M = 1.92, SD = 0.66); t_(22)_ = −2.7459, p = 0.0118, suggesting that students improved their knowledge of core concepts of electronics and neuroscience. The post-survey questions that students had the most difficulty in describing were the role of the synapse (Q2), followed by the transistor (Q3), and then finally information encoding (Q1).

## Discussion

As the Blackawton Primary School has demonstrated [23], K-12 students are capable of not just following experimental protocols, but also participating in scientific discovery. Science provides children a venue to transform the way they think of the world, and scientific inquiry may play an important role in their cognitive development [16], [17], [24]. Early exposure to science provides the potential to excite students and change the world in unexpected ways. William Gates' experience with a PDP-8 in high school eventually led to significant progress in computer science [25]. A 4-inch telescope purchased for a ten-year-old Thomas Bopp eventually led to the discovery of the great comet of 1997 [26]. Exposing students to neuroscience at an early age could provide inspiration to future engineers and scientists to take on the field's greatest challenges.

The reactions of the students and teachers to the workshops were positive; however, improvements can be made. The teachers in our study expressed that the software used to visualize the neural activity should include analysis techniques. Features such as spike-sorting, autocorrelograms, and perievent histograms would be useful for teaching higher-order thinking of neurophysiology. We are currently working to develop the software and lesson plans to support this. Another noted weakness was that our amplifier was susceptible to line noise interference (60 Hz or 50 Hz, depending on global location). If a student's laptop or oscilloscope is plugged into a wall outlet, the SpikerBox can become unusable. It is therefore recommended that demonstrations be restricted to battery-powered devices, though we have found simple faraday cages built with hardware store components for <$20 can reduce much or all of the line noise [18].

While the SpikerBox is loud enough for individual use, it is too quiet for large classroom demonstrations. The spiking activity from a cockroach leg registers at an audible 78.6 dB SPL at a distance of 5.0 mm away from the speaker. Standing even 0.5 m away, however, lowers the sound output to 59.6 dB SPL (SD 0.6), which is too quiet for a large group. Thus, secondary audio amplification with a portable amplifier/speaker (RadioShack part# 277-1008) is sometimes needed. This represents an area for improvement. We are actively researching ways to reduce feedback noise at high amplification so the SpikerBox's internal speaker will be sufficient for classroom environments.

We are not the first to develop low cost amplifiers. Before the first commercial neural recording products appeared, it was common practice for neuroscientists to build their own equipment. While labs typically only used their homebuilt amplifiers internally, some groups have formally written up their designs [27], [28]. Most notably, the CRAWDAD group at Cornell developed a low-cost extracellular amplifier [29] that can be easily built and has similar performance to the SpikerBox (Figure S1).

We found that after the lecture and experiments, students still struggled to explain the role of synapses. This was not surprising as our experiments only indirectly demonstrated synaptic activity through neuropharmacological manipulation. This lies in contrast to the effective demonstration of neural firing (through our rate coding and temperature) where the responses scored much higher. We are continually trying to develop ways to demonstrate the role of synapses. Students also demonstrated difficulty explaining the role of a transistor. Future lesson plans are underway to make the teaching of transistors, op-amps, and other electronics more interactive. For example, we engage the students in a discussion about the role of each part as they solder it to their boards, but we do not discuss the internal components of each part. In future workshops, we can point out that chips contain many transistors arranged in unique ways to do specific tasks.

The two most common questions from students during the workshops were: 1) “Is the cockroach leg still alive?” and 2) “Can the legs grow back?” We explained that so long as the leg is still generating spikes, the leg is indeed still alive. We have also observed leg regrowth and are now systematically studying this phenomenon in controlled experiments.

At the close of our workshops, we asked students to suggest an experiment they would like us to design. We received two prominent answers: “the effect of drugs on the brain” and “neuroprosthetics”. Since we had already shown one neuropharmacological experiment, this response indicates a request for more extensive (perhaps illicit) pharmacology. Due to the restricted nature of neuroactive compounds, finding off-the-shelf ways to demonstrate neuropharmacology remains a challenge. The neuroprosthetic experiment request, however, we were able to accommodate. We recently designed an experiment in which we connect the neural output of one leg into another leg. When one leg is brushed with a toothpick, the other leg moves. This experiment and others are described fully online [18], and we will continue to update the content based on feedback from our field work in classrooms. As our SpikerBoxes continue to be used as a teaching tool by many scientists and educators, we foresee in the years to come the development of compelling experiments that students themselves imagine.

## Supporting Information

Figure S1 **Comparison of the SpikerBox with Other Amplifiers.** (a) Recording traces made with the iPhone application. (b) Histograms of iPhone recordings used to calculate SNR. SNR was defined as the number of standard deviations (Z-Score) away from the mean the spike peaks were in the histograms (arrows). The SpikerBox has a built-in gain of 900, band-passed from 300 to 1300 Hz. The Cornell amp has a built-in gain of 1000, band-passed from 160 Hz to 5 kHz. The SRS560 was set as closely equivalent to the SpikerBox as possible, with a gain of 1000 and band-passed from 300 Hz to 1 kHz.
(TIFF)

File S1 **Detailed Circuit Diagram and Teacher Guide for Building a SpikerBox.**
(DOCX)

File S2 **Student Hand-Out for Experiment I - How do neurons carry information about touch?**
(DOCX)

File S3 **Student Hand-Out for Experiment II - How do neurons generate electricity?**
(DOCX)

File S4 **Student Hand-Out for Experiment III - How does your brain tell your muscles to move?**
(DOCX)

File S5 **Student Hand-Out for Experiment IV - How do drugs affect neurons?**
(DOCX)

File S6 **CadSoft Eagle .sch file (circuit layout) for printing SpikerBox Circuit.**
(SCH)

File S7 **CadSoft Eagle .brd file (board layout) for printing SpikerBox Circuit.**
(BRD)

File S8 **CadSoft Eagle .lbr file (parts library) for printing SpikerBox Circuit.**
(LBR)

File S9 **Excel Spreadsheet of Parts/Costs of SpikerBox components with associated part numbers from electronics supplier digikey.com.**
(XLSX)
