# Supplementary figures and images for: The SpikerBox: A Low Cost, Open-Source BioAmplifier for Increasing Public Participation in Neuroscience Inquiry
Source: PLoS One. 2012 Mar 21;7(3):e30837. doi: 10.1371/journal.pone.0030837 (PMC3310049; doi:10.1371/journal.pone.0030837)

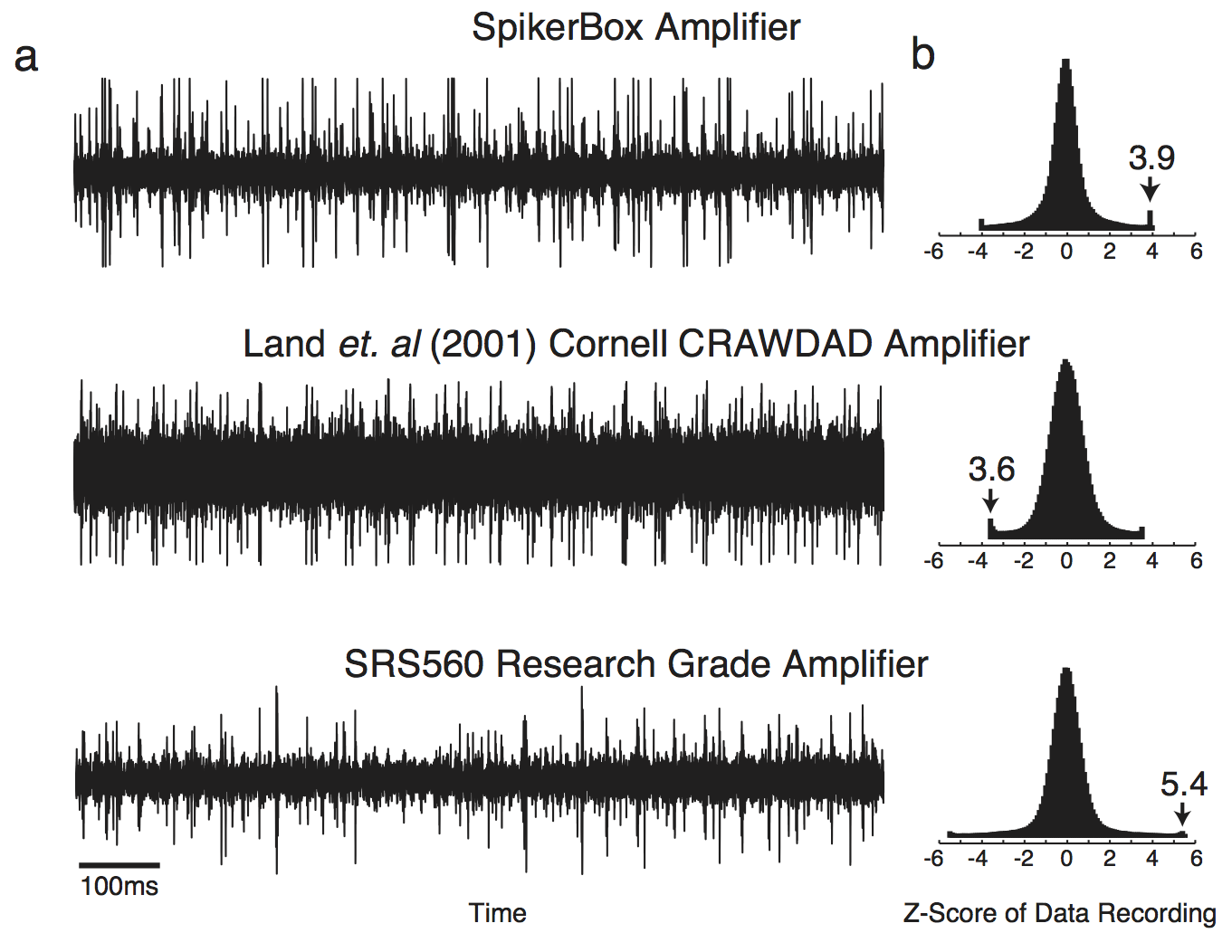

Supplement: Figure S1 — Comparison of the SpikerBox with Other Amplifiers. (a) Recording traces made with the iPhone application. (b) Histograms of iPhone recordings used to calculate SNR. SNR was defined as the number of standard deviations (Z-Score) away from the mean the spike peaks were in the histograms (arrows). The SpikerBox has a built-in gain of 900, band-passed from 300 to 1300 Hz. The Cornell amp has a built-in gain of 1000, band-passed from 160 Hz to 5 kHz. The SRS560 was set as closely equivalent to the SpikerBox as possible, with a gain of 1000 and band-passed from 300 Hz to 1 kHz. (TIFF) [file pone.0030837.s001.tiff]
