# Supplementary material for: The SpikerBox: A Low Cost, Open-Source BioAmplifier for Increasing Public Participation in Neuroscience Inquiry
Source: PLoS One. 2012 Mar 21;7(3):e30837. doi: 10.1371/journal.pone.0030837 (PMC3310049; doi:10.1371/journal.pone.0030837)
Supplement: File S1 — Detailed Circuit Diagram and Teacher Guide for Building a SpikerBox. (DOCX) [file pone.0030837.s002.docx]

**Detailed Circuit Diagram and Teacher Guide for Building a SpikerBox**

So why do we even need a SpikerBox to listen to neurons? Neurons communicate using action potentials (“spikes”) that have very low electric current (I). To visualize this activity, we can use electrodes (in our case stainless steel needles) which have a high impedance (resistance). The pins we use have a 1 kHz impedance typically around 20-30 kΩ. A low current with high resistance can produce a voltage large enough that we can use (V=R*I). We can continue to amplify the signal in stages to be able to see and hear the neural signals. Let’s take a look at how this is done.

The invention of the transistor (by Bell Labs in the 1940s) ushered in a big change for electronics. A transistor allows a signal to become amplified (louder) through the use of semi-conducting materials such as silicon. This invention is considered to be one of the greatest of the 20th century, as it has allowed computers to get very small and very fast. In the SpikerBox, we use transistors to amplify the spikes many, many times.

Below is an annotated circuit diagram (or schematic) of the SpikerBox. Viewing a circuit diagram is a lot like viewing a subway map. The topology (connections) is important, while the geographical location can be changed to make the topology easier to view.


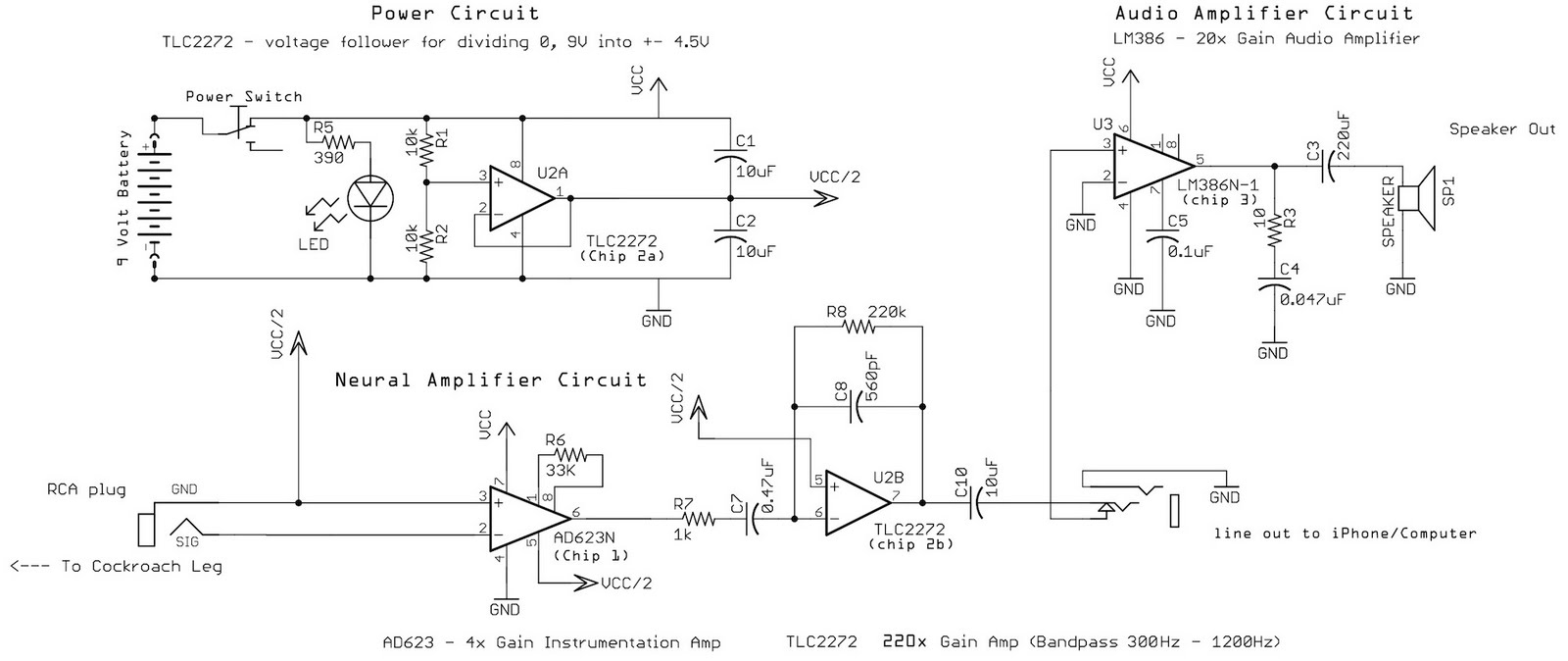


**Power Circuit.** Operational amplifiers (op-amps) are symbolized by a right pointing triangle and are used to amplify the difference between the input pins. Op-Amps are made up of 20-30 transistors connected together to allow this special type of amplification. Typically, op amps use dual-supply voltages (V+,V-) which allow the inputs and outputs to be referenced to ground (0 V). This would require 2 batteries (one each for V+ V-). For portability, we designed our circuit to use a single 9 V battery, and we therefore split the voltage into ±4.5 V using a voltage divider (R1/R1). The virtual ground is stabilized by an op-amp (Chip 2a) using a voltage follower. We used a TLC2272 as our op-amp, but similar parts could be used from other suppliers (TL074, OP291, OP293, MCP602).

**Neural Amplification Circuit.** The two pins from the cockroach leg enter the circuit from the RCA plug on the left. This signal is amplified ~4x by the AD623 low-voltage instrumentation amp (Chip 1). An instrumentation amp is a special type of differential amplifier which is particularly suitable for use in measurement applications. You can also use an INA118 chip from Texas Instruments instead of the AD623. The gain (amplification) is set by adjusting the resistor across pins 1 and 8 according to the equation:

Gain = 1 + (100kΩ/R)

In our circuit, R6 = 33kΩ, thus the gain is 4.03. The output of the AD623 is further amplified through a second TLC2272 op-amp (chip 2b), with the equation:

R8/R7 = 220kΩ/1kΩ = 220

This section of circuit also eliminates (filters out) frequencies that we are not interested in. The neural spike signals have frequencies of~500-1000 Hz in waveshape, so we designed the filter to allow those through. The resistor and capacitor in series (C7 and R7) serve as a high-pass filter with a cut-off frequency determined by f=1/(2πRC). Our high pass cut-off is thus 338 Hz. The resistor and capacitor in parallel (C8 and R8) serve as the low-pass filter, with a cut-off frequency determined by the same equation, and thus is 1291 Hz. The total gain of the circuit is 4.03*220 = 886.6. This amplified signal then goes to the line out on the SpikerBox that can then be connected to a computer using a patch audio cable (RadioShack part #42-2420) or to an iPhone using a custom iPhone cable (easy to build for ~$9 in parts, see wiki.backyardbrains.com).

**Audio Amplifier Circuit.** The current build of the circuit includes an audio amplifier (using the LM386, chip 3) and a built-in speaker for listening to neural activity. The LM386 is configured to amplify the signal an additional 20x using the standard setup on the freely available LM386 data sheet from National Semiconductor.

**Low Frequency Version of SpikerBox.** To modify the SpikerBox bandpass settings to allow low frequency electrophysiological signals such as electromyograms (EMGs) and/or local field potentials (LFPs), C7 (capacitor 7) can be changed to 10 uF, and C8 can be changed to 4.7nF. These are commonly available capacitor values; in this set-up, the bandpass of the SpikerBox is now 15.9 - 153 Hz; the gain remains unchanged.

##### Building Instructions

SpikerBox kits are available for teachers to assemble in the classroom. The diagram below shows a physical description of all the required parts. Alternatively, parts can be ordered from online suppliers. A full list of parts, and their associated Digi-Key part numbers, are available in supporting file S9. We have also supplied our printed circuit board (PCB) schematic and board layout in Eagle (CadSoft; Oberhaching, Germany) format in supporting files S6-S8.


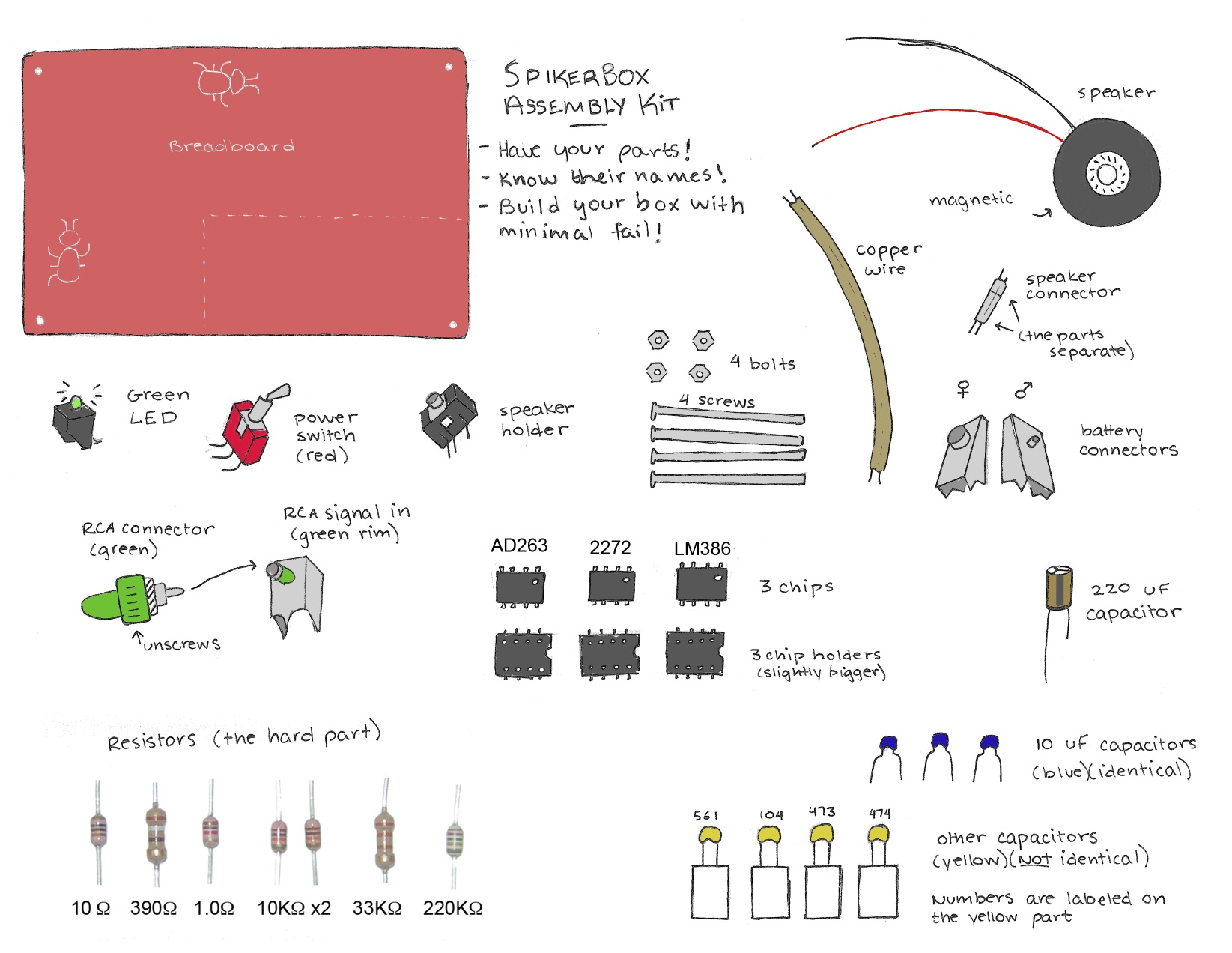


##### Materials Needed:

Below are list of things needed to get you started. A Soldering Iron can be purchased at a local hardware store. The Magnifying Glass and Silly Putty are available at drug stores.

1. Soldering Iron
2. Solder
3. Magnifying Glass to read labels on Chips and Capacitors
4. 9V battery to power your SpikerBox.
5. Silly Putty to hold components in place on board while you solder on backside
6. Pair of scissors
7. Wire Strippers and Wire Clippers
8. Sandpaper to allow the speaker to friction fit into enclosure.
9. Electrical Tape

###### SpikerBox Circuit Construction:

So long as the placement is correct, you can solder components in any order. We suggest that the parts be soldered starting from the row of connectors, to the chip holders, to the components. The chips should be the last thing placed on the board. Accompanying photographs of every stage of construction can viewed at diy.backyardbrains.com

1. Solder Speaker Connector.
2. Install Audio Line Out.
3. Install Power Switch.
4. Install LED (bulb pointing out).
5. Install RCA signal in.
6. Install Chip Holders. The half circle should point toward the front of the board (where input, switch, LED, and line out are).
7. Install 220 uF capacitor (the big one) at C3. **Grey Stripe (minus for negative) needs to point toward connector row.**
8. Install 10 uF (blue cap) capacitor at C10, C1, and C2.
9. Install 10 kΩ resistors at R1 and R2.
10. Install 390 Ω resistor at R5 (near LED).
11. Install 33 kΩ resistor at R6 (near RCA signal in).
12. Install 1 kΩ resistor at R7,
13. Install 0.47 uF capacitor at C7. The capacitor has “474” in small text on it.
14. Install 220 kΩ resistor at R8.
15. Install 560 pF capacitor at C8. The capacitor has “561” in small text on it.
16. Install 0.047 uF capacitor at C4. The capacitor has “473” in small text on it.
17. Install 10 Ω resistor at R3.
18. Install 0.1 uF capacitor at C5. The capacitor has “104” in small text on it.
19. Install the 9V battery connectors. Take care to ensure the two connectors are flush with each other or else battery will not plug in. **Take care that the positive and negative terminals are correctly positioned (check your battery).** The “skinny” connector (male) goes on the bottom of the board. We recommend soldering with a battery in place.
20. Install Chips in holders, with circle on all chips pointing towards top of board. AD623 goes in left holder, LM386 on right holder, and the TLC2272 goes in the middle holder.
21. Install battery.

###### Enclosure Construction:

1. Place screws in bottom enclosure piece, with screws facing up.
2. Slide your completed SpikerBox over the four screws.
3. Cut vinyl spacers into four equal lengths.
4. Place four vinyl spacers on screws.
5. Solder Speaker Connector (female) on ends of speaker wire. Take care that the two solder joints do not touch. Separate and wrap solder joints away from each other using electrical tape.
6. Press speaker in hole on top enclosure, you may need to sand it a bit.
7. Plug Speaker connection into SpikerBox Circuit Board
8. Slide top enclosure piece on screws.
9. Secure construction by installing nuts on exposed ends of screws.
10. Add cork stickers (stack them) to have a place to stick the specimen.

######

###### Electrode Construction:

1. Unscrew end of RCA connector.
2. Split and Strip ½ inch lengths from speaker wire on both sides and both ends.
3. Place wire in eyelets of RCA connector, solder, and crimp where applicable. Take care that connections are both strong and not touching each other.
4. Rescrew RCA end back on connector.
5. Solder two sewing needles on other ends of speaker wire.
