# Supplementary material for: The SpikerBox: A Low Cost, Open-Source BioAmplifier for Increasing Public Participation in Neuroscience Inquiry
Source: PLoS One. 2012 Mar 21;7(3):e30837. doi: 10.1371/journal.pone.0030837 (PMC3310049; doi:10.1371/journal.pone.0030837)
Supplement: File S2 — Student Hand-Out for Experiment I - How do neurons carry information about touch? (DOCX) [file pone.0030837.s003.docx]

**Experiment I**

**How do nerves carry information about touch?**

**Background:**


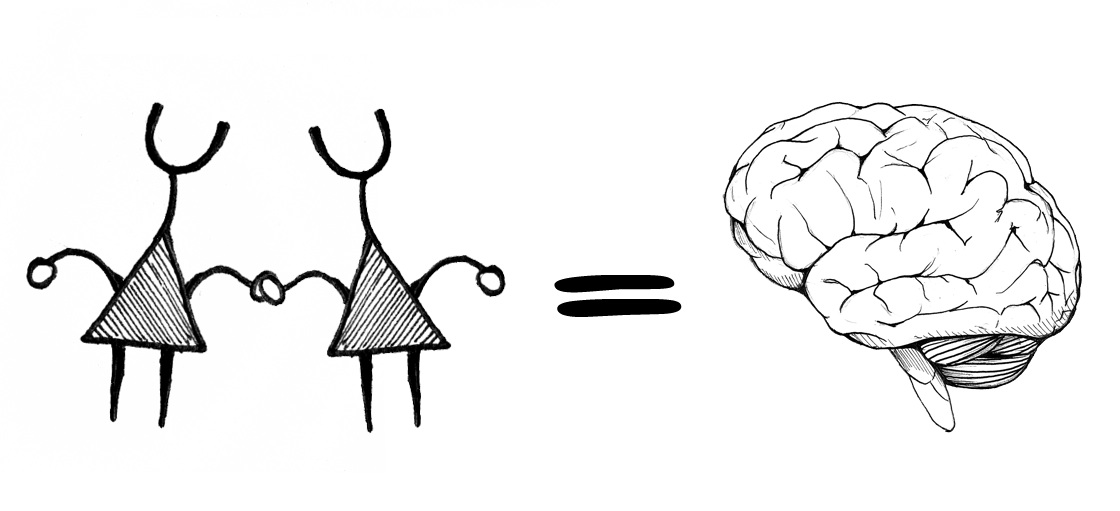
Your brain uses a combination of chemicals and electricity to operate. Brain Cells (neurons) need to communicate with each other to control your body. A brain with only 1 neuron is not a brain.


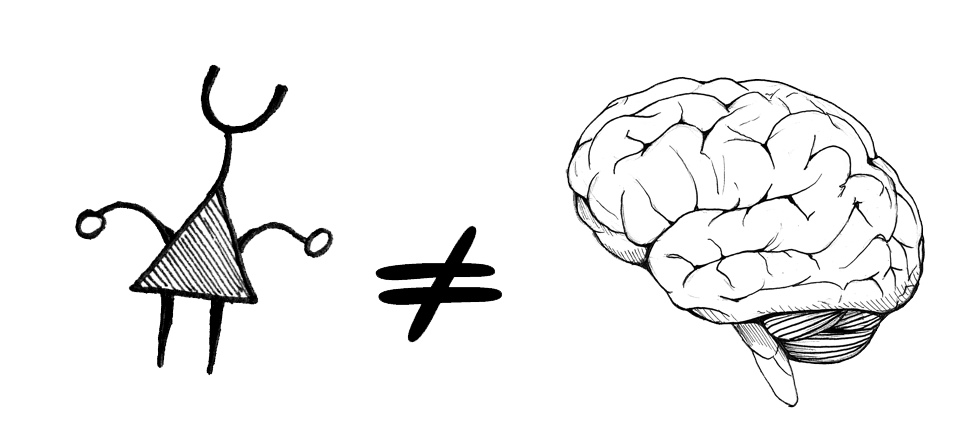


A brain is a network (friendship) of neurons. But how do neurons talk to each other? One of the first ways cells used to network was chemical communication.


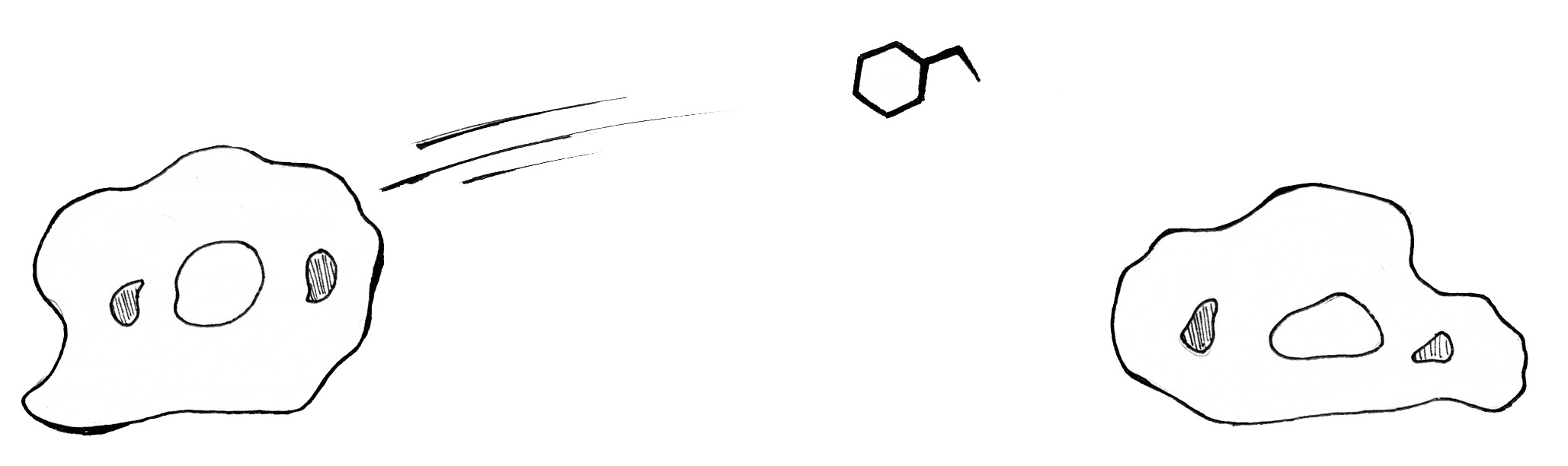


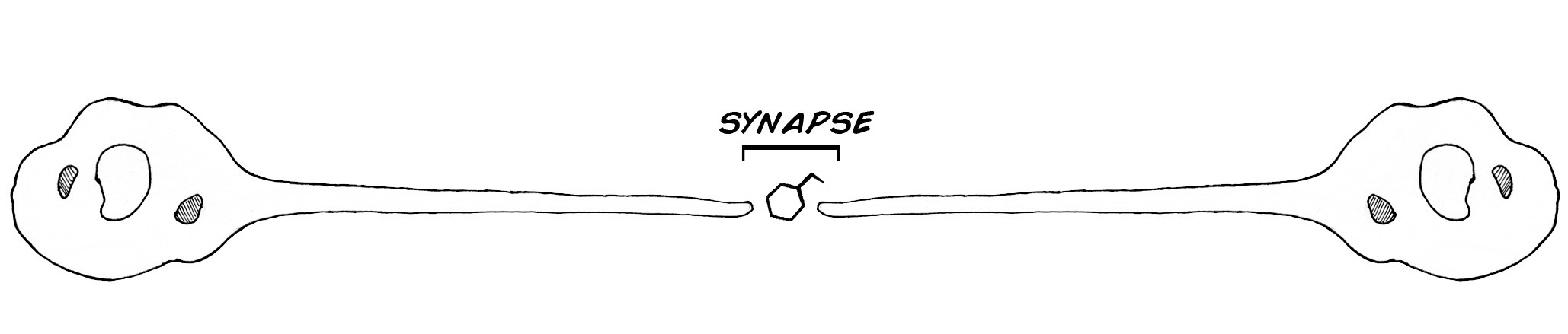
Bacteria use this method. It works well, but is limited by diffusion. For example, when you fart, how long does it take for someone on the edge of the room to smell it? There should be a faster way. One way is to bring cells closer together through stretching.


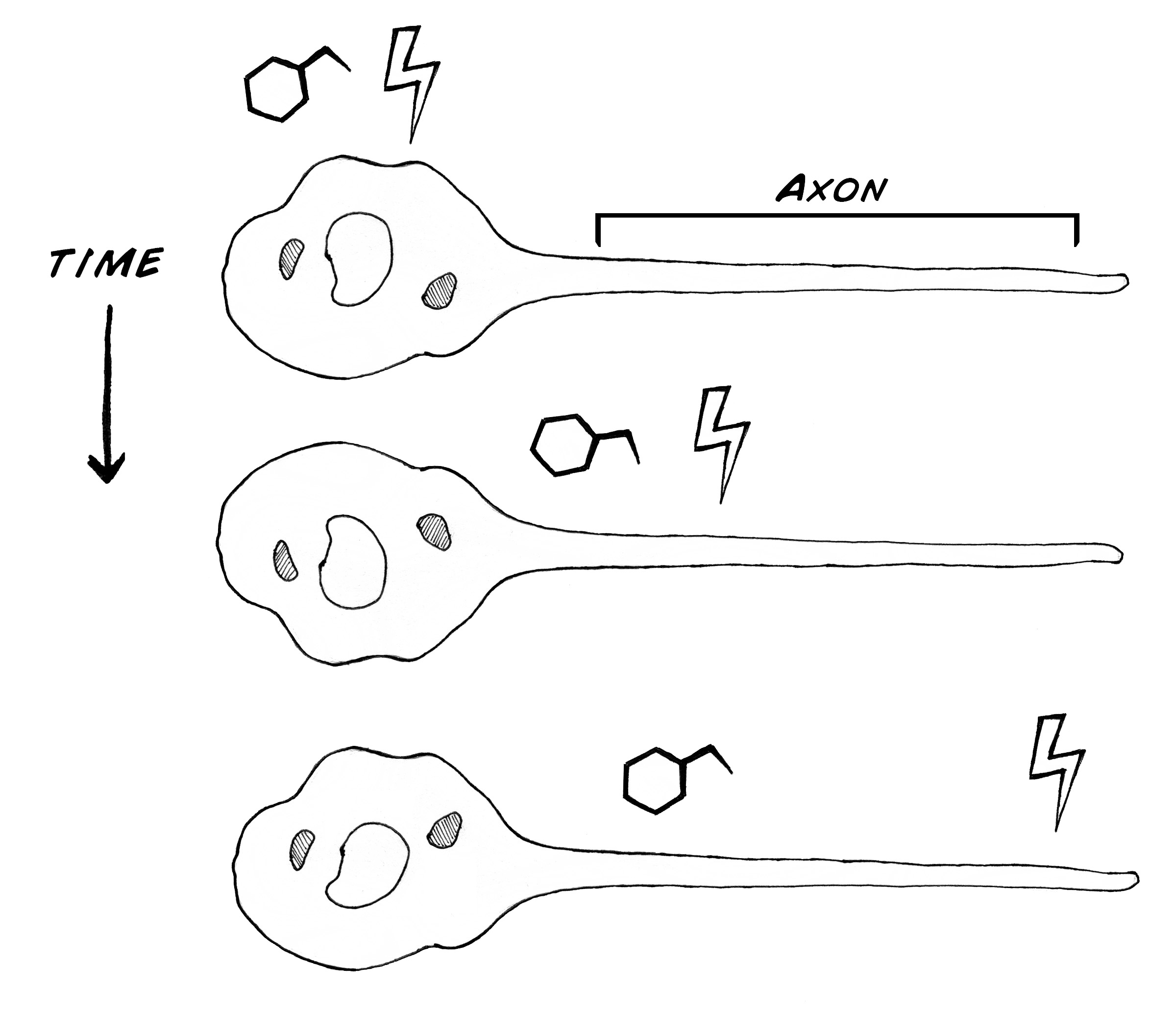


But there is still a problem. The signal still needs to travel a long way through the cell. Is there a way to make this faster? What is very fast & important today?

Electricity! Notice how fast the lights in your house turn on when you flick the switch. Neurons use electricity as well; electrical pulses travel down the neurons. This pulse is called the……………


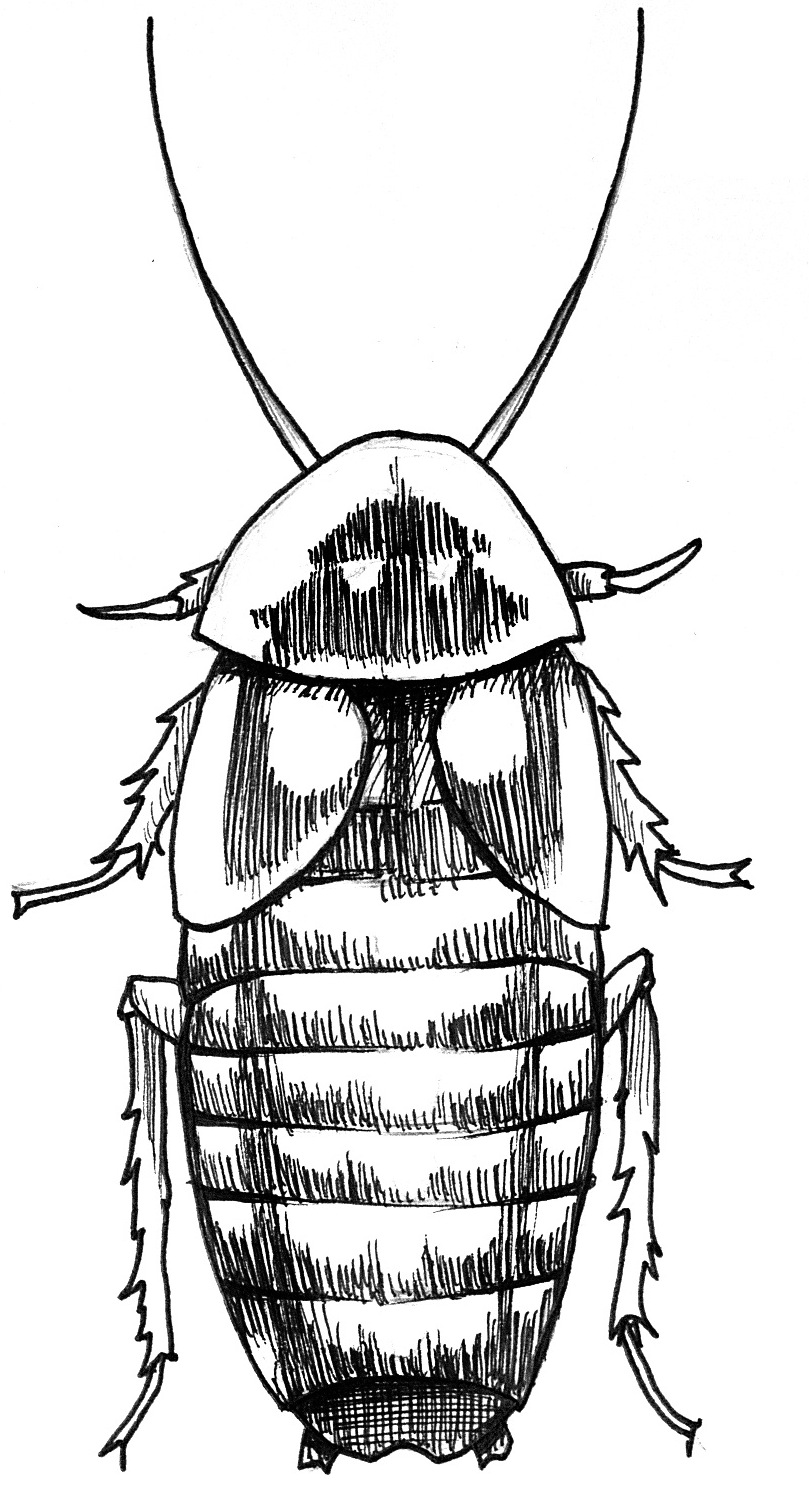

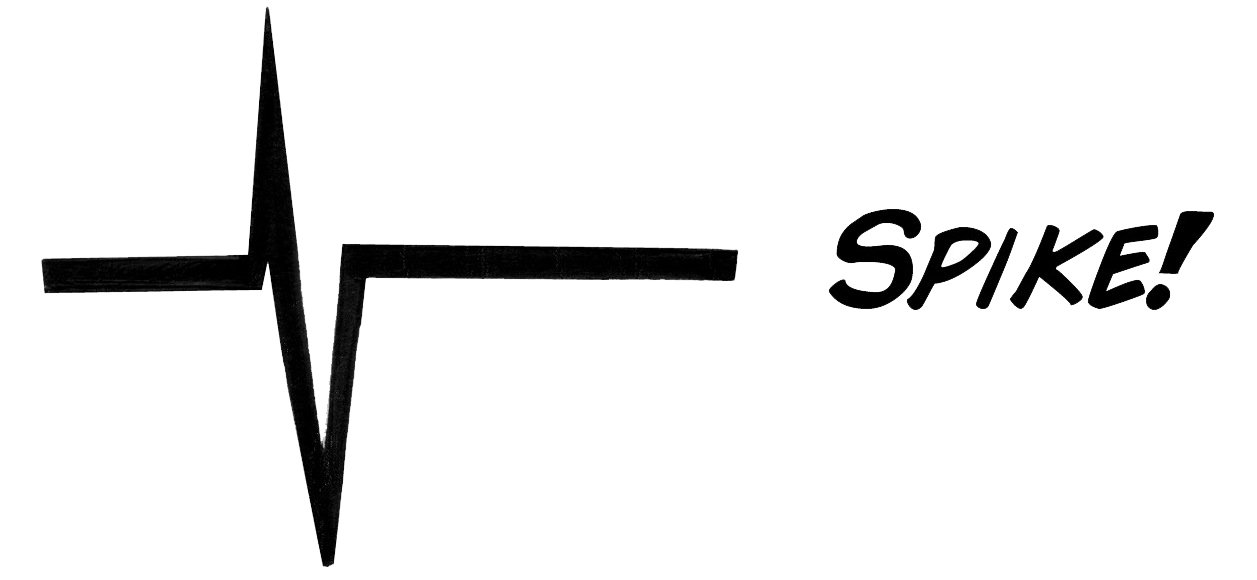
We at Backyard Brains have dedicated our lives to studying spikes, and you can too! But first, some biology. 380 million years of evolution bring you the cockroach. We will use the Discoid cockroach (*Blaberus discoidalis*), or false death’s head. They live in the Amazon rainforest of South America under the bark of rotting trees.

Like all animals (beyond creatures like sea sponges), cockroaches’ bodies are filled with nerves to control movement & sensation, among many other things. Let’s begin.

**Procedure:**

1. Take a cockroach & put it in a jar of ice water. Wait a few minutes until it stops moving.
2.
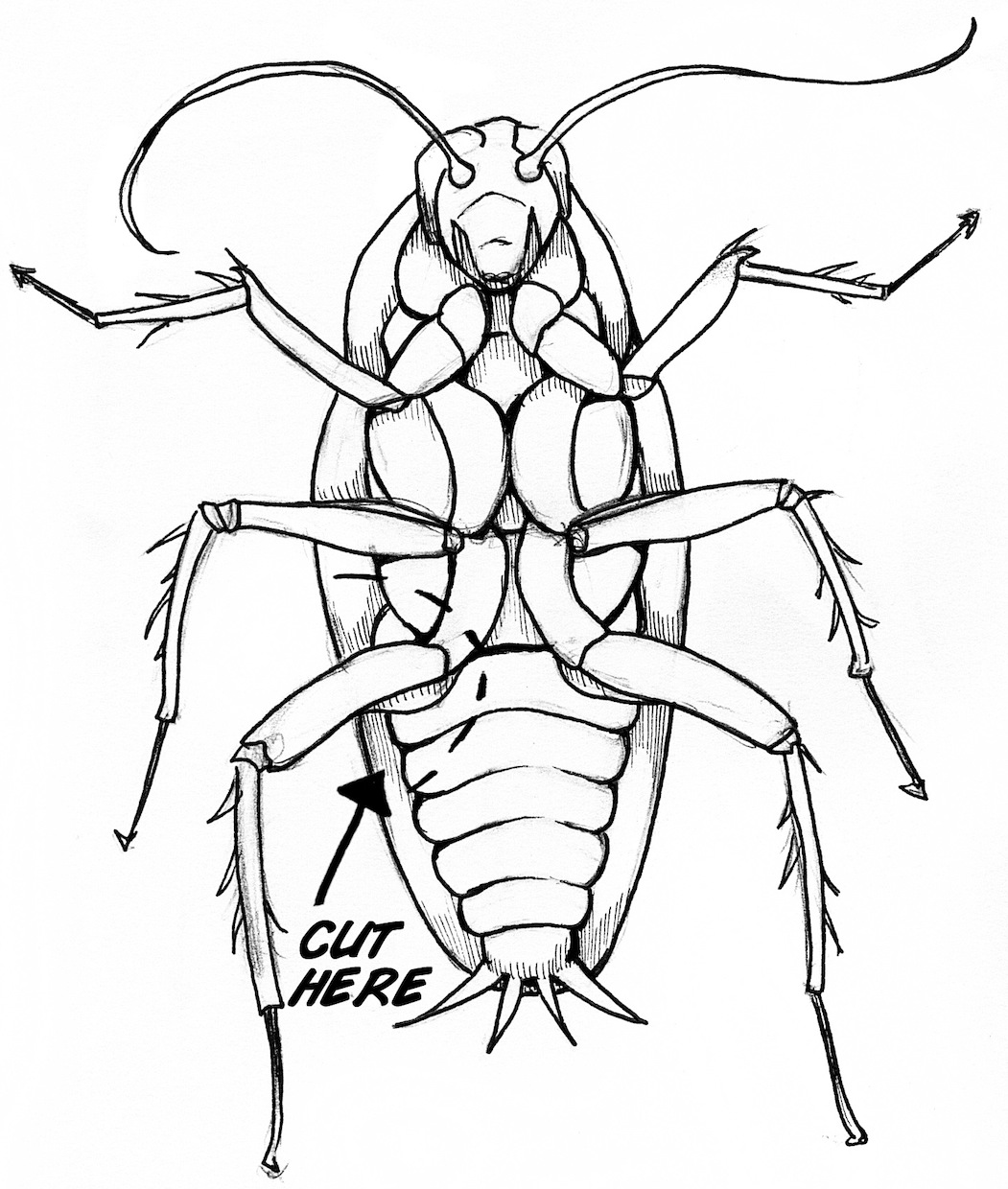
Remove the cockroach, and cut off one of his legs near the body, so that you end up with a leg like this:


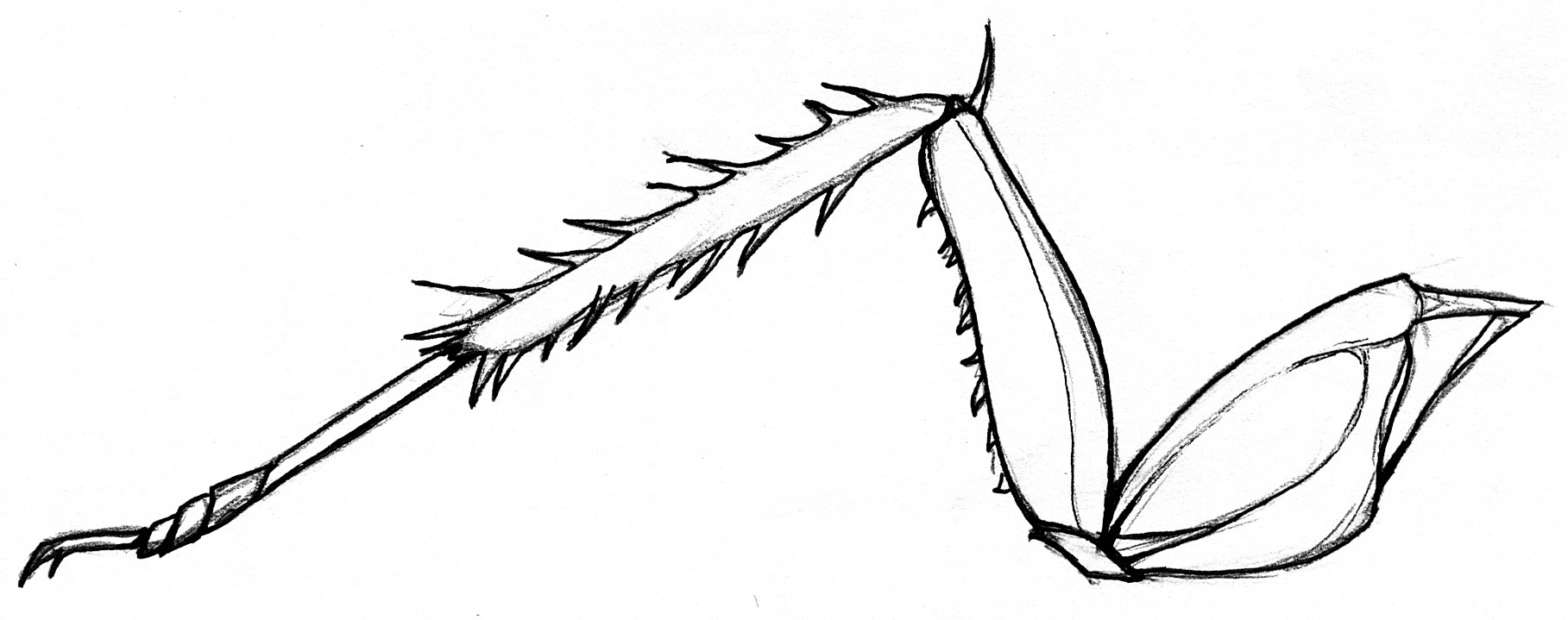


1. Return Cockroach to its house. It'll be fine; the leg will grow back if the cockroach is not a full grown adult yet (Adults have wings, nymphs don't).
2.
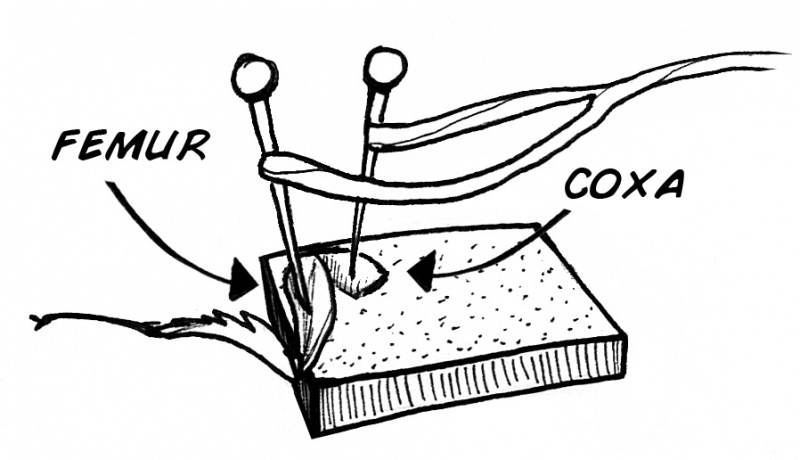

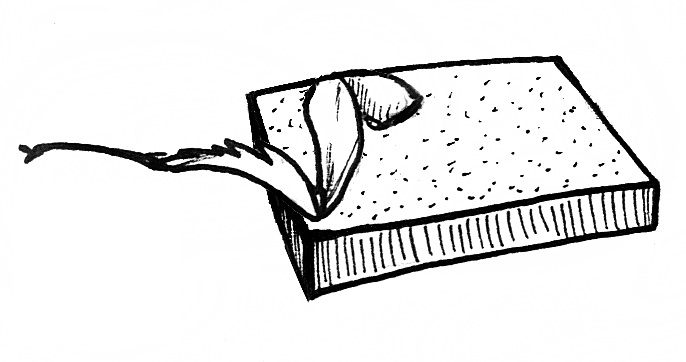
Place the leg on the cork of your SpikerBox, allowing a bit of the leg to overhang, and put the two electrodes in:
3. Turn your SpikerBox on! If you hear a popcorn sound, congratulations, you have just heard your first neuron!
4. Now let's see what the electrical discharge looks like. Plug your cable from the SpikerBox into your iPhone or into the microphone input of your computer. Turn on “Backyard Brains” (iPhone/Android) or Audacity (laptop). You should see:


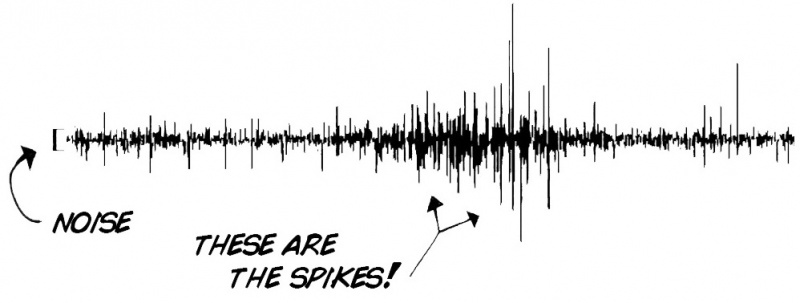


You’ve heard the spikes; but what do they do? The spikes are a way of neurons communicating with each other. The simplest way is with rate, or number of spikes per second. We will discover this property of neurons today. What you need are your breath and a toothpick.

**Background:**

Cockroaches’ legs are covered with barbs that can detect wind and other movements, much like your cats’ whiskers. These barbs tell the cockroach whether the wind is blowing, how fast, and in what direction! Let’s do a simple experiment.

**Procedure:**

1. Turn on the SpikerBox with the leg attached like you did in the last experiment.
2. Blow lightly on the leg. Did you hear anything?
3. Blow vigorously on the leg. Is there any difference?
4. Each thorn on the leg is innervated by a nerve. The more the thorn moves, the more the neuron fires! This is called “rate-coding.”


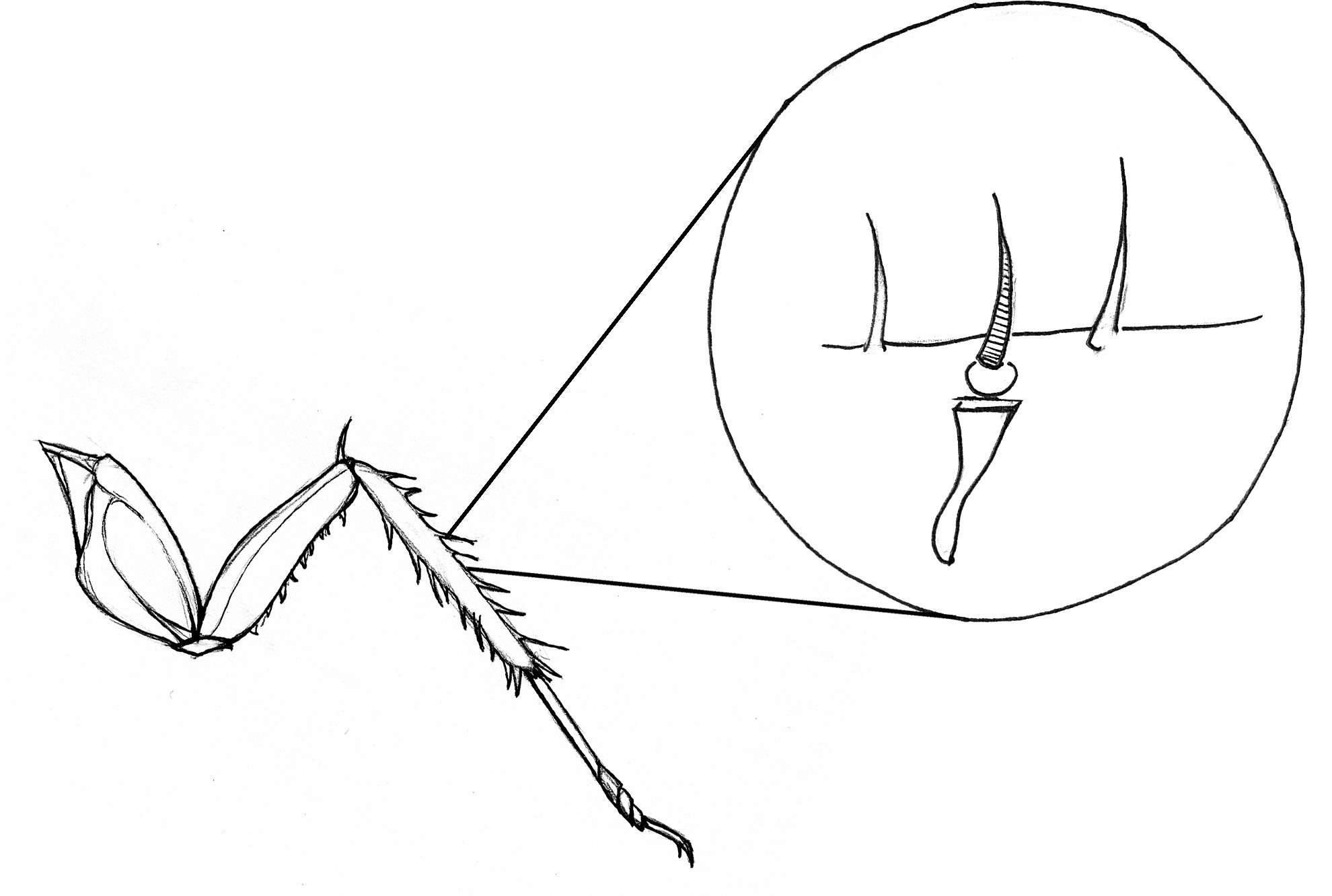
Let’s take another look with a method that is easier to control than your breath. Taking your small toothpick, lightly touch each barb until you find one that causes the loudest, most vigorous change in spikes. Touch the barb in different directions, with varying degrees of pressure. Do you notice anything?
