# Supplementary material for: The SpikerBox: A Low Cost, Open-Source BioAmplifier for Increasing Public Participation in Neuroscience Inquiry
Source: PLoS One. 2012 Mar 21;7(3):e30837. doi: 10.1371/journal.pone.0030837 (PMC3310049; doi:10.1371/journal.pone.0030837)
Supplement: File S3 — Student Hand-Out for Experiment II - How do neurons generate electricity? (DOCX) [file pone.0030837.s004.docx]

**Experiment II**

**How do neurons generate electricity?**

**Background:**


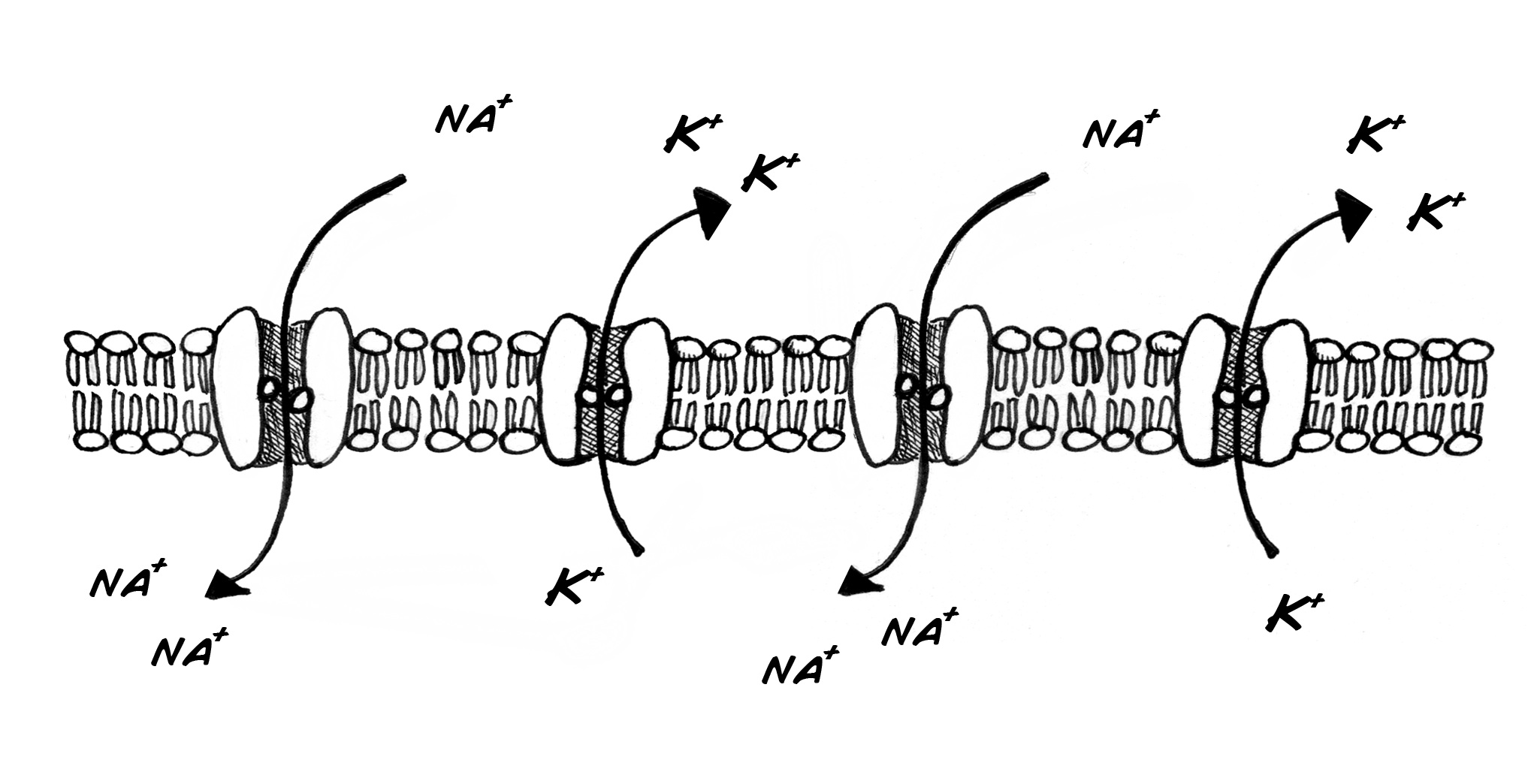
The electrical activity you are observing in the cockroach leg is caused by special proteins called ion channels. These ion channels transport sodium (Na+, in table salt) and potassium (K+, in bananas) across the neural membrane to generate the spike. Using a sophisticated microscope not invented yet, the neural membrane would look like this:

The delicate dance of the sodium and potassium ion channels is what causes the change in electrical activity. Building and maintaining these pumps requires energy (produced from the food creatures eat) and the right temperature. If the temperature is too low, the channels will not open, and electrical pulses cannot travel down the nerve.


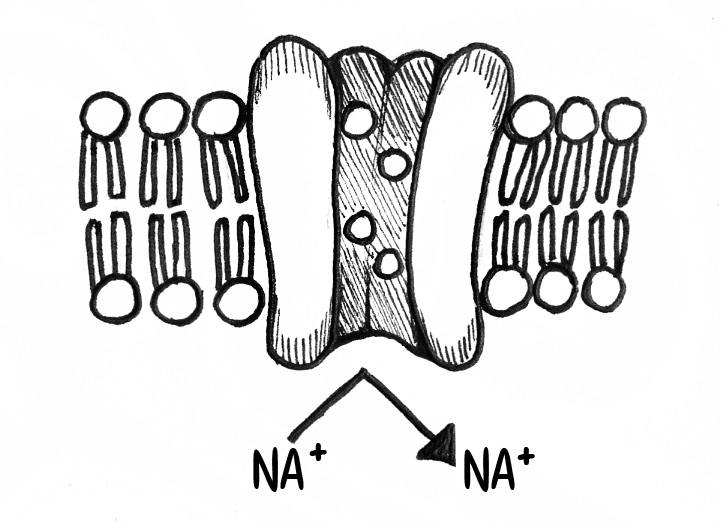


If the temperature is too high, the ion channels & nerve membranes come apart.
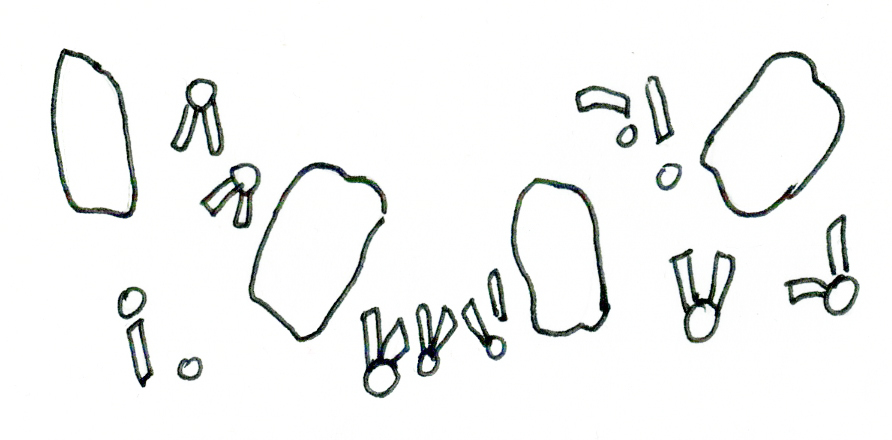


**Procedure:**

We can observe these two processes indirectly with the cockroach leg prep. Take your SpikerBox, and place it in the freezer of a household refrigerator. Make sure the cable is long enough to go from your laptop into the freezer, and stays connected to the SpikerBox. Also place a digital thermometer inside the freezer, and wait and listen for 1-3 minutes….


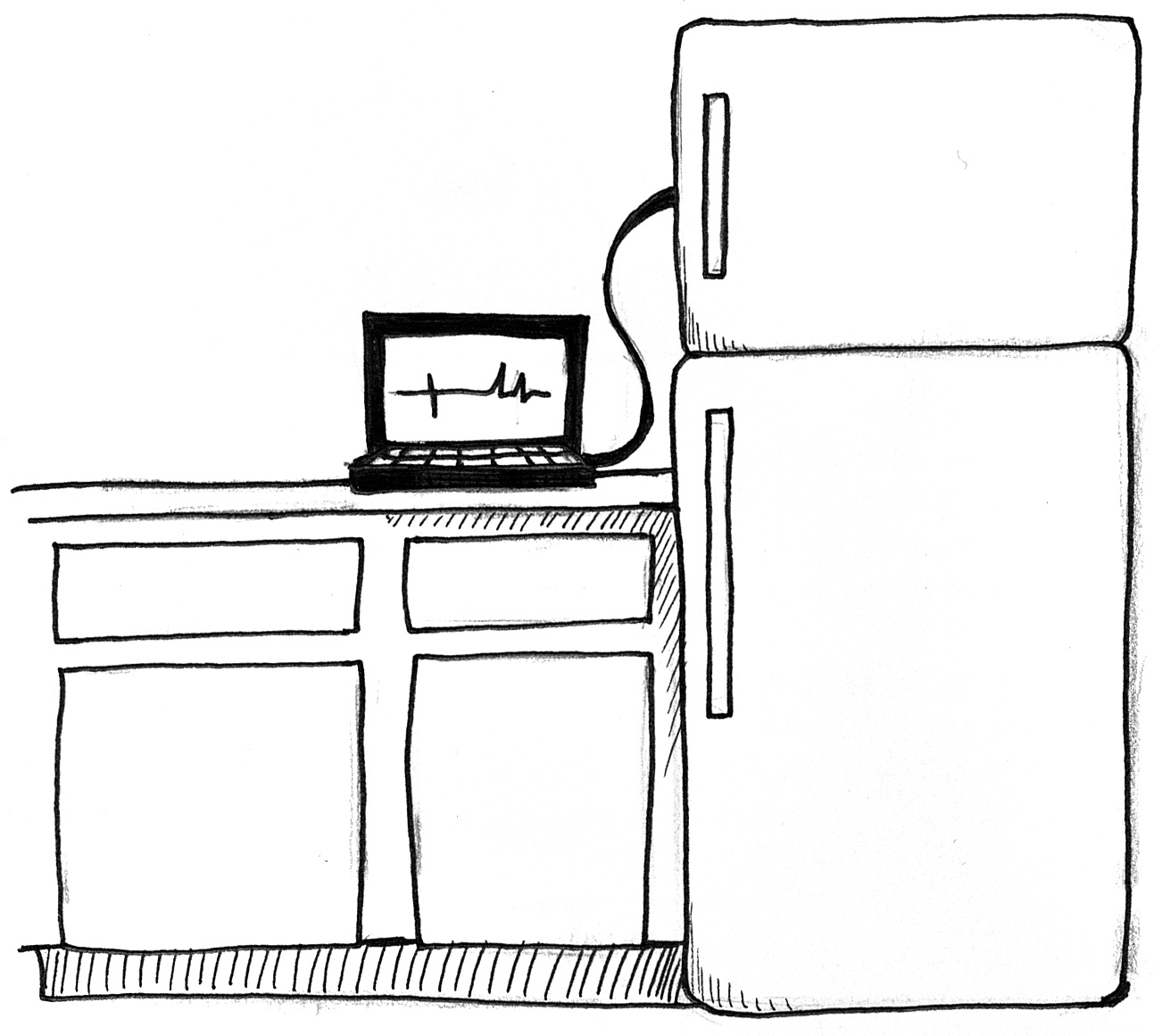


At a certain temperature, the spikes should go away. Open the freezer & record the temperature inside with a thermometer. Once you take the SpikerBox out of the freezer, how long does it take for the spikes to come back?

Note: depending on your freezer model, you may find a lot of electrical noise occurring when you put the SpikerBox in it. If you live in cold land like Michigan, you can simply take your SpikerBox outside in deep winter. Otherwise, if you have access to dry ice (solid carbon dioxide, usually available at ice cream stores), you can put the dry ice and the SpikerBox in a cooler. This method is noise-free, as it does not use electricity!

We will not reveal the temperature at which spikes go away, as that is for you to find out, but we will give you a hint: normal ice, which is typically 31-33 degrees Fahrenheit, is not cold enough. If you use water ice instead of dry ice in your cooler, your spikes will not go away.

Now let’s try in the opposite temperature. You need

1. A candle
2.
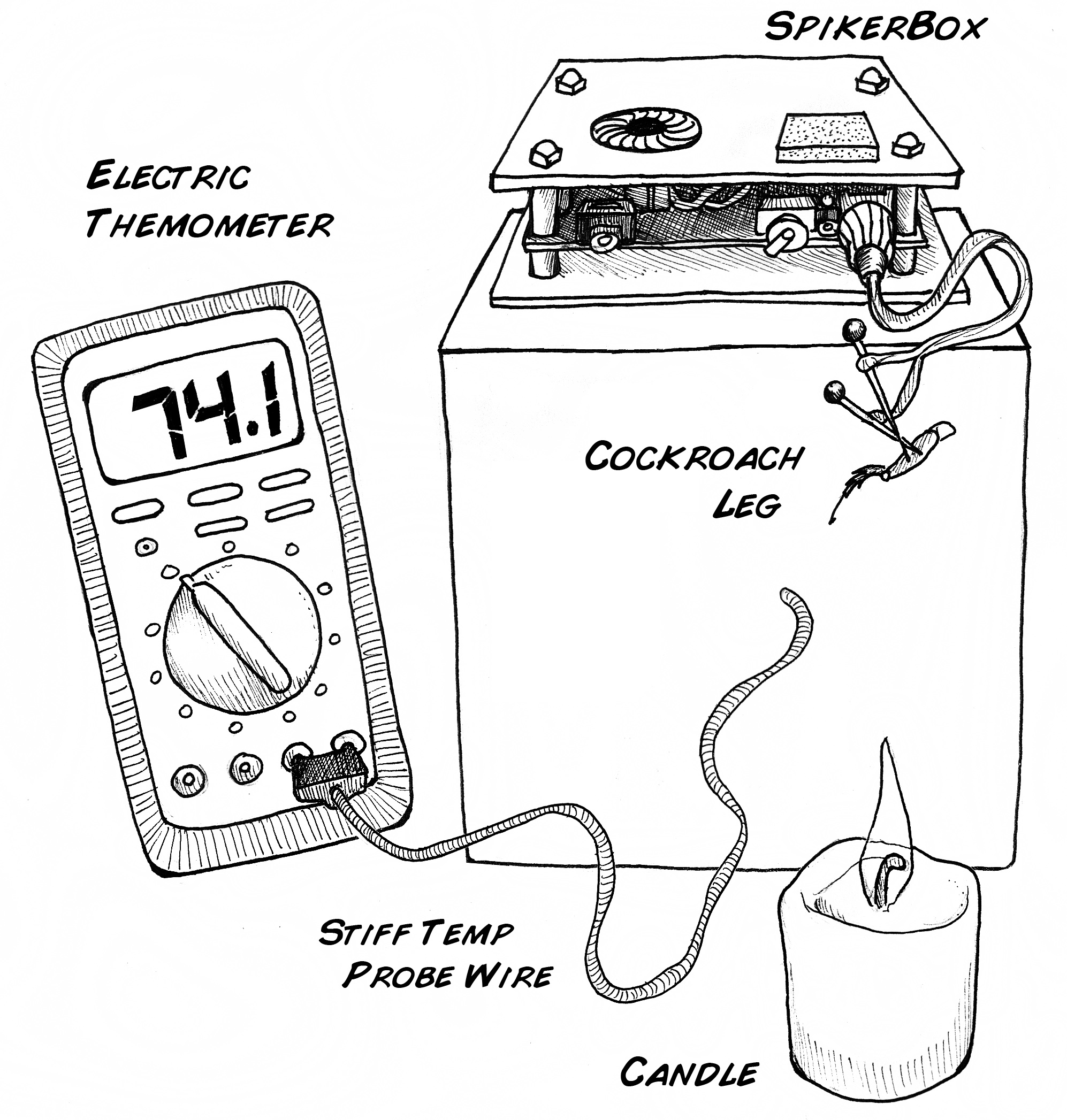
A box approximately 5-6 inches taller than the candle

Place the SpikerBox on top of the box, and have the electrode with the cockroach leg attached hanging out “in space” 5 to 6 inches over the top of the candle. Position the wire of your digital thermometer such that you can measure the precise temperature where the leg is. Now, to quote hero Alan Shepard, “light this candle!” As the temperature slowly rises, you should hear the neurons responding, and at a certain temperature, you will hear a very low “scream” of spikes as all the neurons discharge. Record the temperature at which this happens, and exhaust the candle. When the leg gets back to room temperature, test to see if the spikes come back. Try this hold/cold experiment with a few legs.


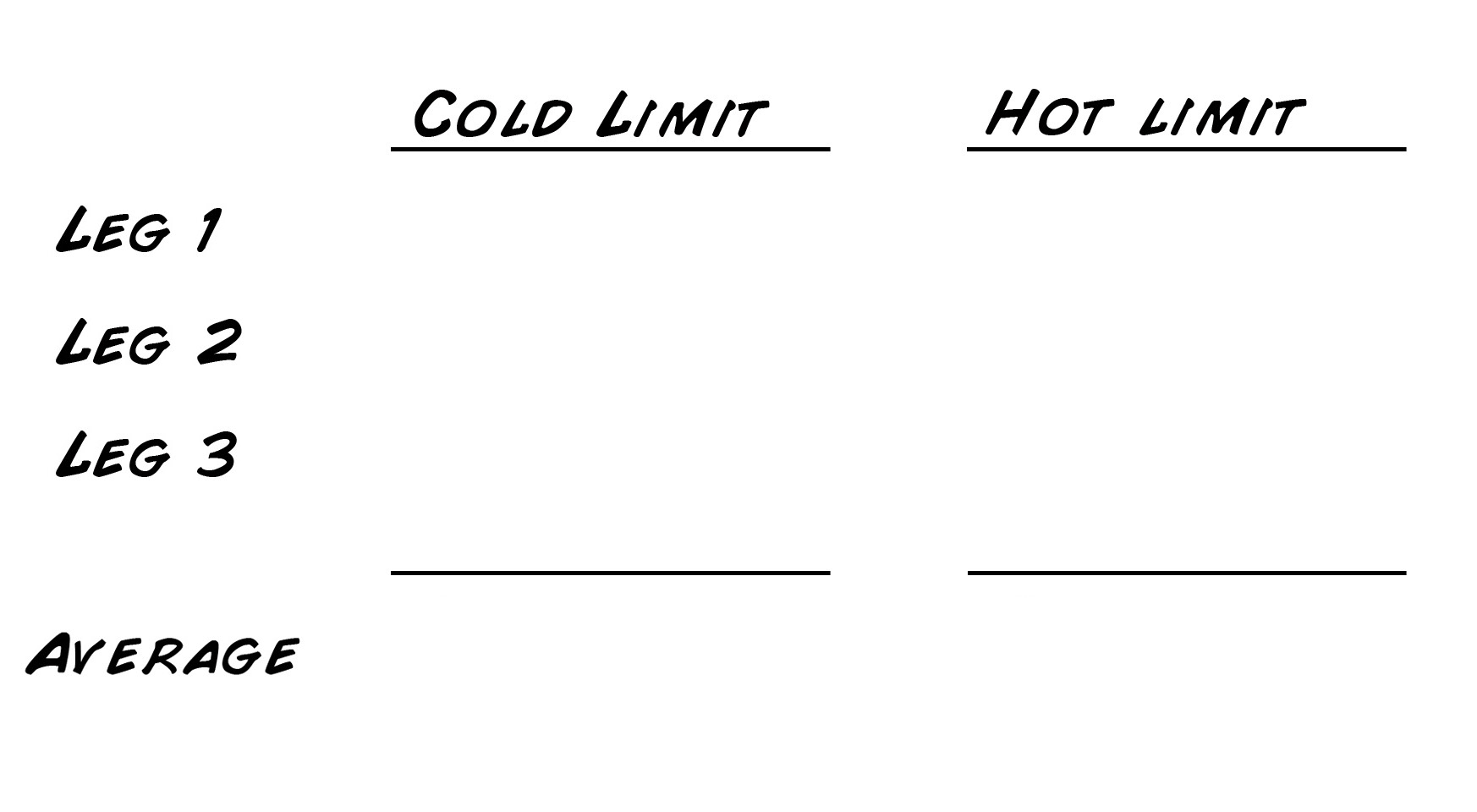
As you know, different insects have different tolerances to heat and cold. Perhaps you should try this experiment with a variety of different legs of different species.
