# Supplementary material for: The SpikerBox: A Low Cost, Open-Source BioAmplifier for Increasing Public Participation in Neuroscience Inquiry
Source: PLoS One. 2012 Mar 21;7(3):e30837. doi: 10.1371/journal.pone.0030837 (PMC3310049; doi:10.1371/journal.pone.0030837)

**Experiment III**

**How does your brain tell your muscles to move?**

**Background:**


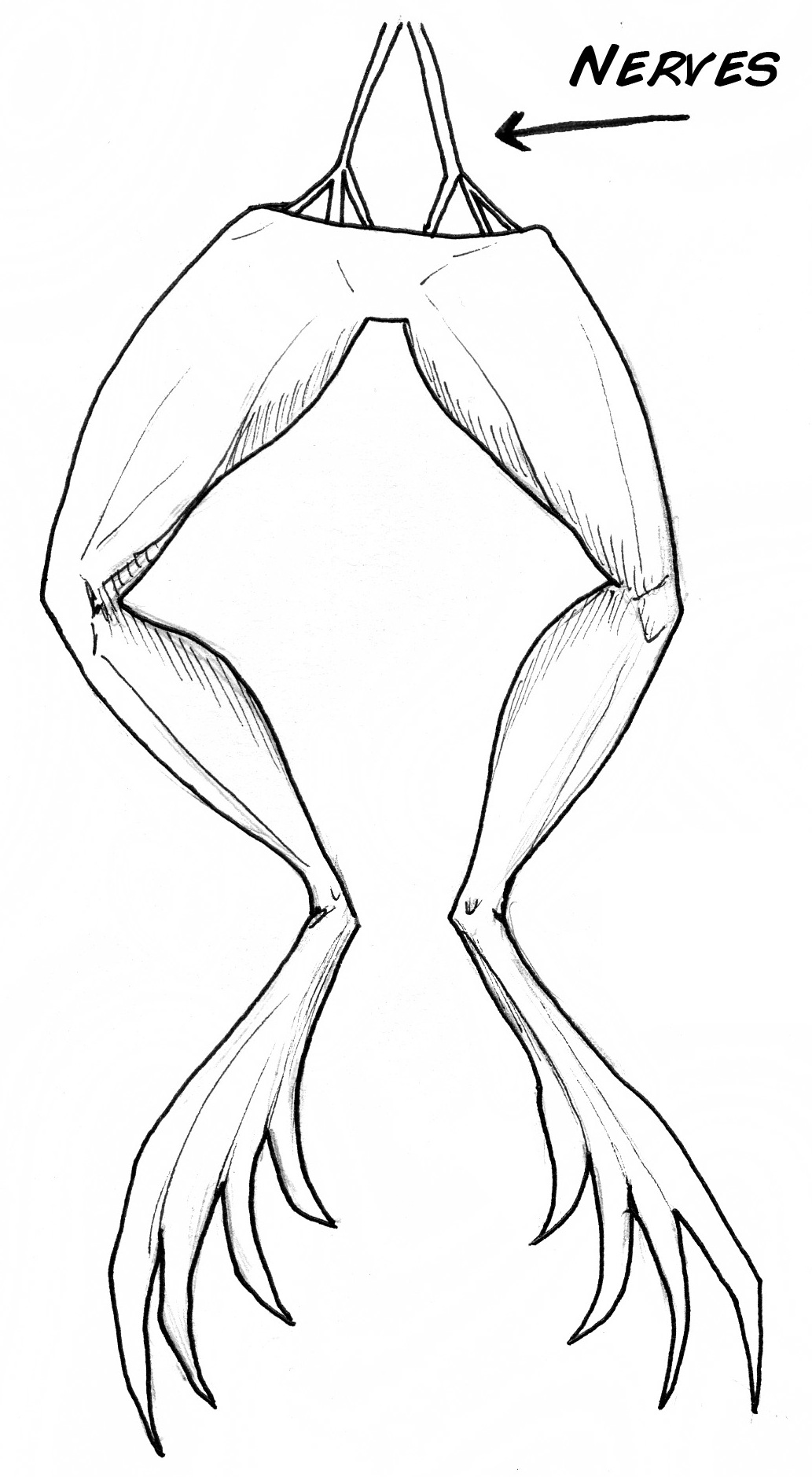
Long before scientists were able to record spikes, they were able to stimulate the nervous system using batteries (Leyden Jars). Since nerves use electricity to communicate, they can be manipulated with electricity as well. Luigi Galvani, an Italian scientist in the 1700’s, discovered that electricity applied to the nerves of frog legs caused the large muscles to twitch.

Such discoveries led to debates at the time as to whether “animal electricity” was different from the electricity during lightning storms. Galvani also tested this by hanging frog legs off his back porch during thunderstorms & watching the legs twitch.


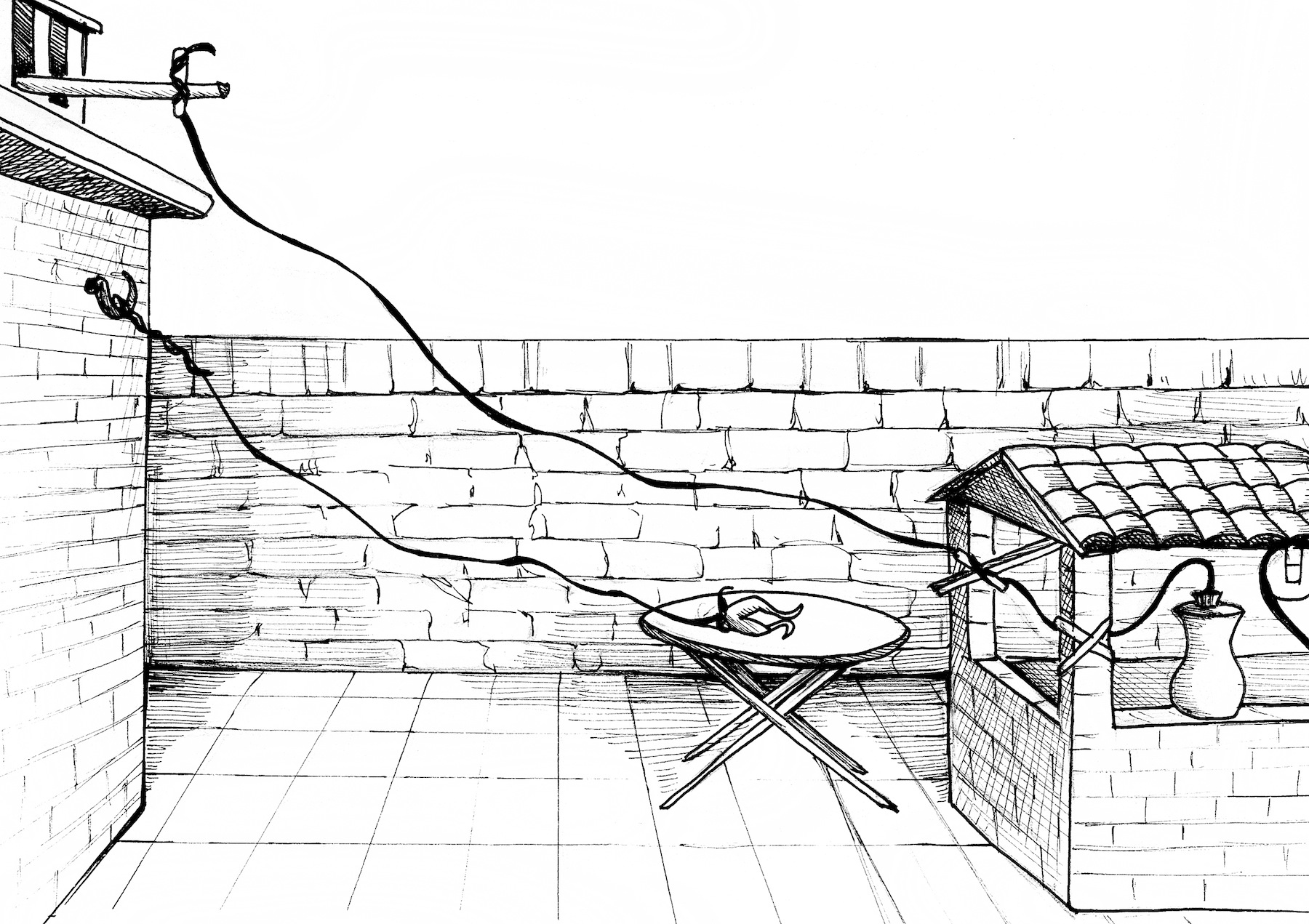
Eventually the scientific community agreed & discovered that while electricity can indeed stimulate nervous system and muscle tissue, the tissue itself generates electricity. This led to the beginnings of contemporary neuroscience which you are studying today.

In another famous experiment, German Medical Scientists Eduard Hitzig and Gustav Fritsch in 1870 applied electricity current to the exposed cerebral cortex (wrinkly part of brain) of dogs in their kitchens (yes, it was odd even back then) showing that stimulation of different parts of the brain can cause different types of movements.


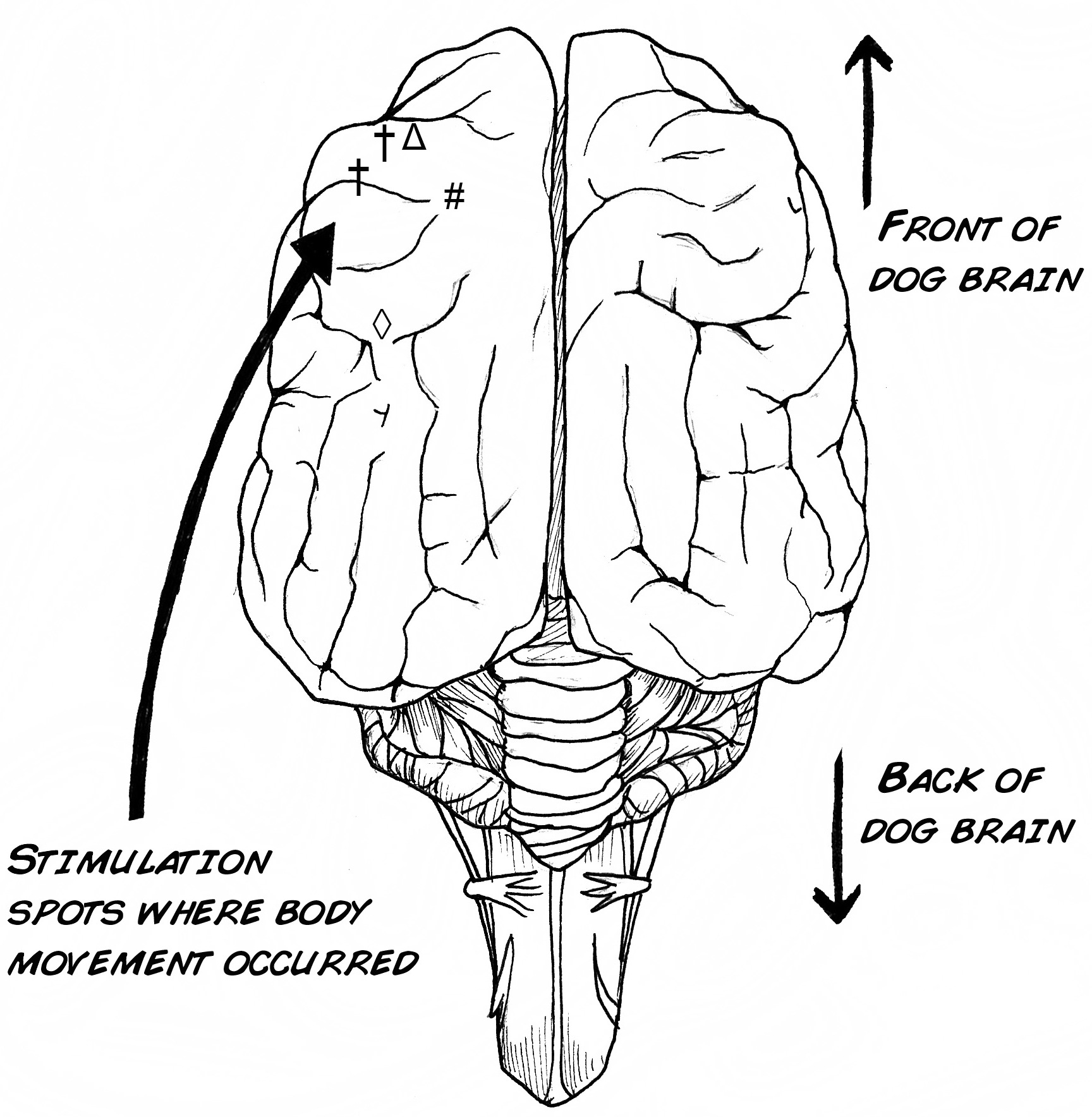


Today, such techniques are used in patients, most notably those afflicted with Parkinson’s disease.

By inserting a small, long electrode into a specific part of the brain called the subthalamic nucleus, the shaking and tremors associated with the disease can be lessened. Sometimes there are side-effects though, like increased gambling and other compulsive behaviors.


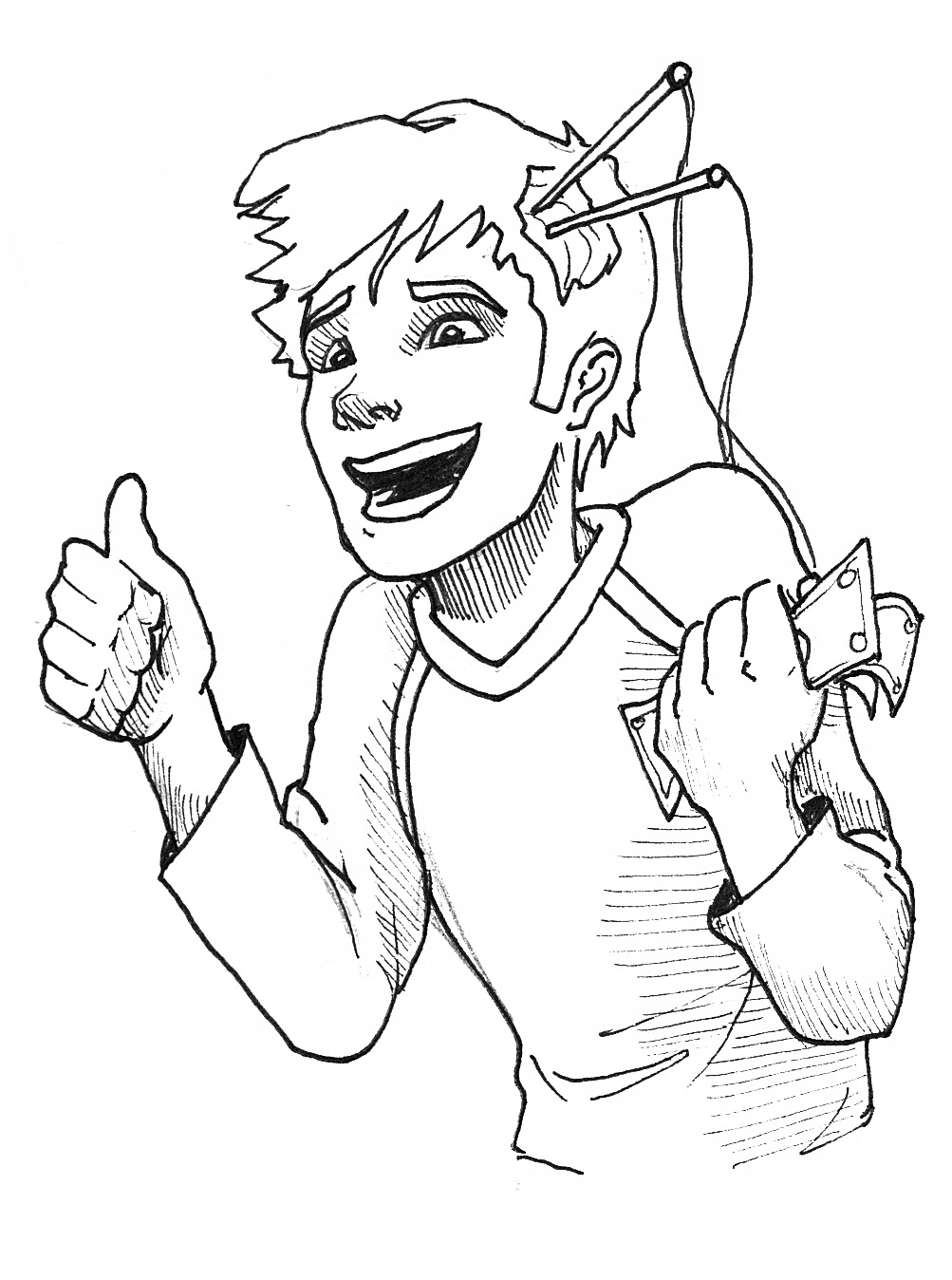
Today, some advanced research groups are designing small chips that stimulate the nerves of the eye as a cure for blindness.


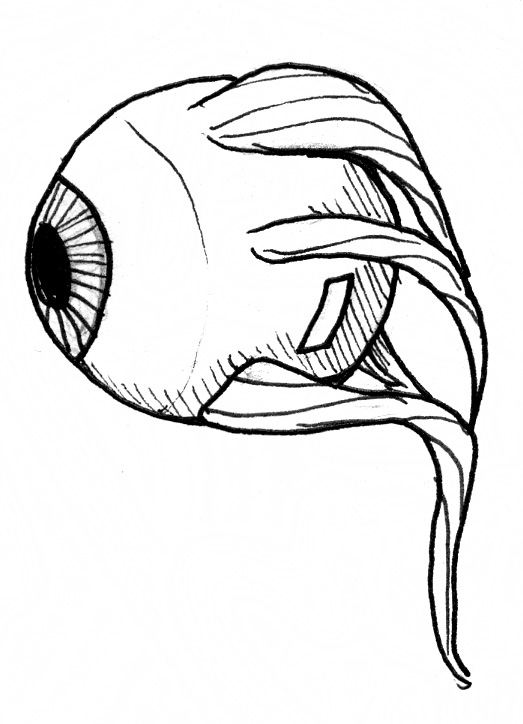


**Procedure:**

Today we will study electrical stimulation in a very simple experiment, using only:


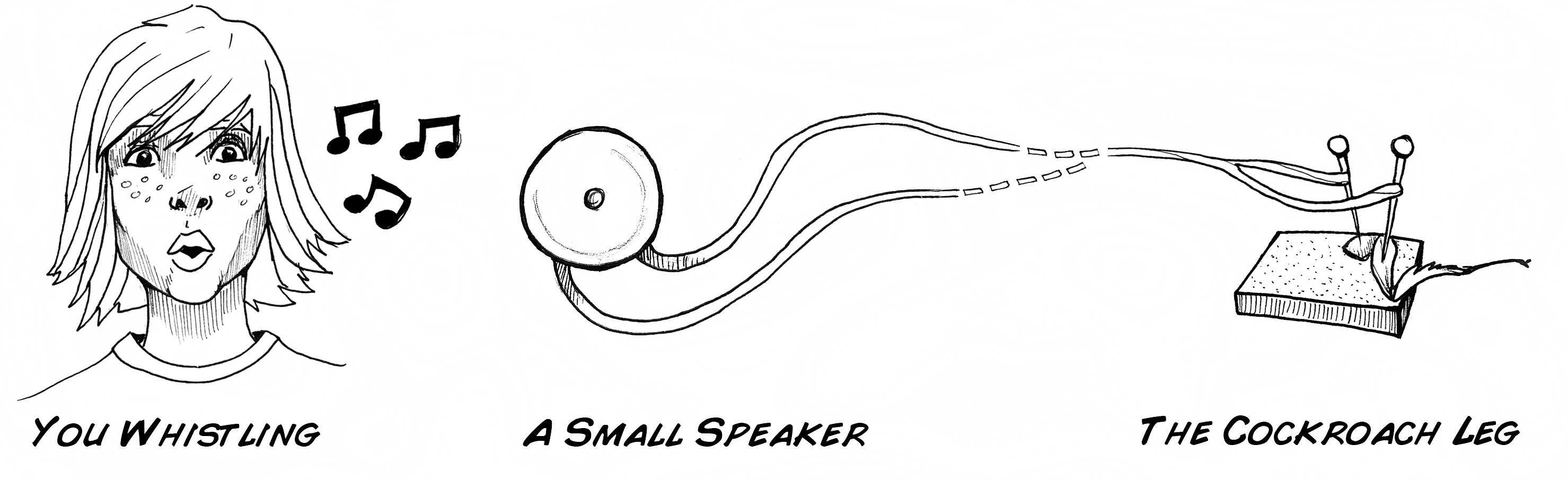


 First, some theory on how speakers work:

The electric current signal in the wires passes through a magnetic field in the speaker, which causes a cone/drum to move, pushing the air and creating the sound that you hear. For example, have you even seen a bass woofer vibrate at a rock concert?


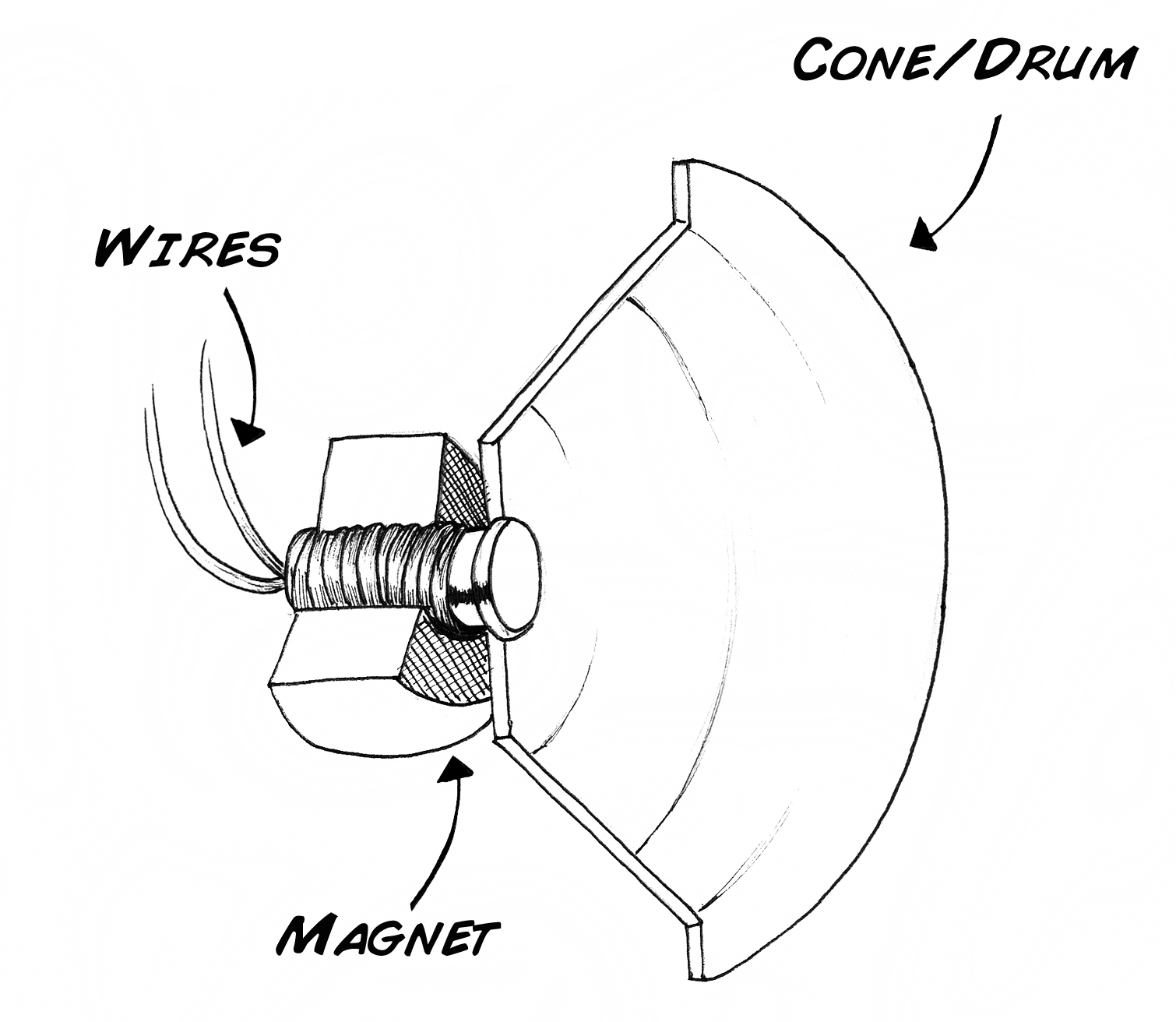
This principle also works in reverse, and this is how microphones work. If you speak into a microphone/speaker, the movement of the cone/drum causes a current to flow in the wires. If we use a special speaker called a [piezoelectric](http://www.radioshack.com/product/index.jsp?productId=2062402), we can generate quite large voltages (1-3 V), large enough to actually excite nervous and muscle tissue!

Connect the two leads of your piezoelectric speaker to the needles in the cockroach leg using your long connector wires, place the speaker close to your mouth, and try to whistle as loud as you can. Watch the leg; as you whistle louder and louder, the leg should begin to move.


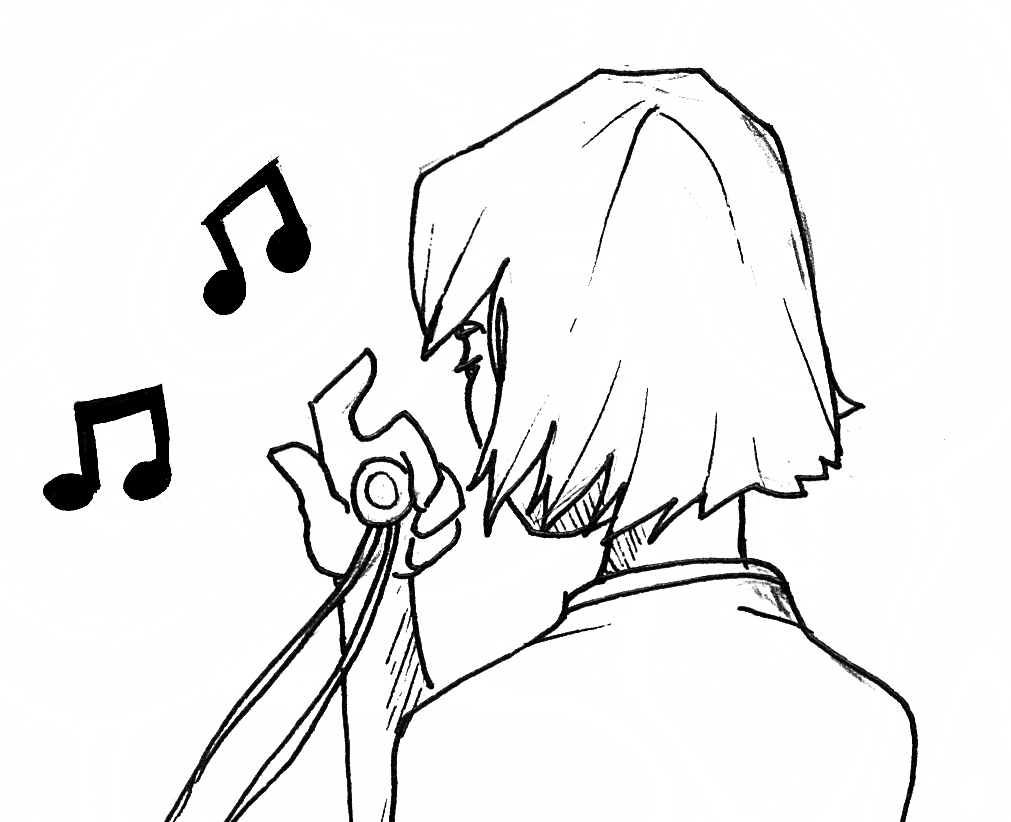
Such phenomenon, when first discovered by Galvani, were the inspiration for Mary Shelley’s “ Frankenstein.”

“Perhaps a corpse would be re-animated; galvanism had given token of such things: perhaps the component parts of a creature might be manufactured, brought together, and endured with vital warmth.”

-Mary Shelley, Introduction to Frankenstein


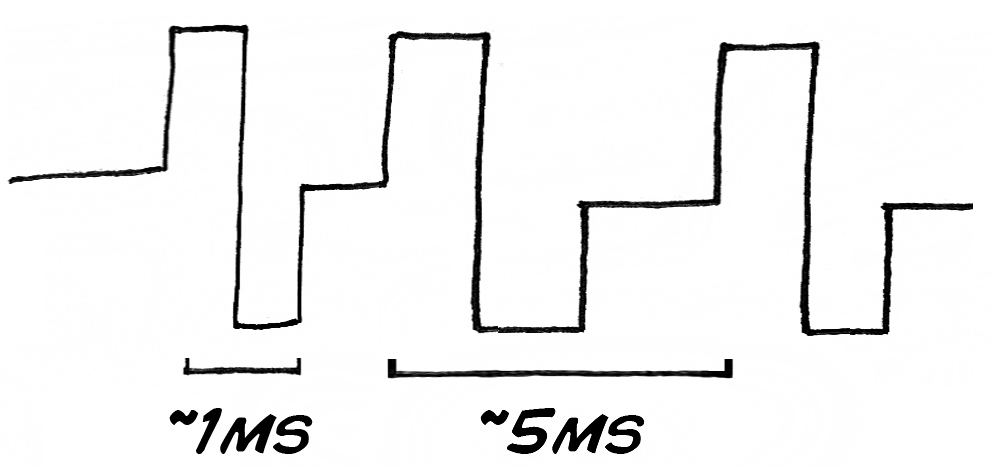

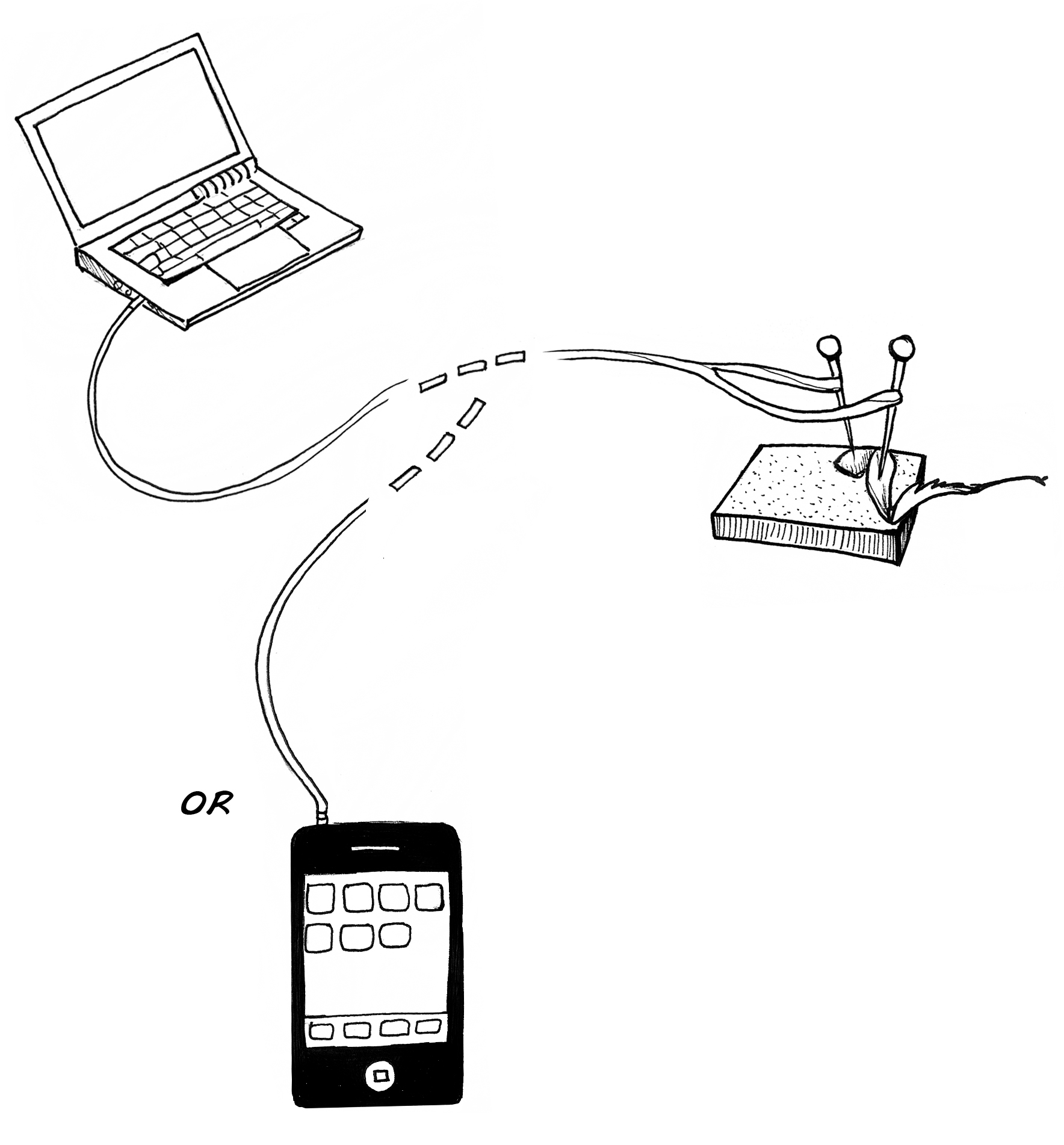
It would be ideal, of course, to have finer control over the stimulation than simply whistling into a speaker. Fear not; there is an easy way to do this. Using applications (which are free) like ToneGen on your laptop, or AudioSigGen or FreqGen on your iPhone, you can control the frequency & amplitude of the stimulus you deliver to the leg. In humans with implanted devices like cochlear implants & deep brain stimulators, the stimulus looks like this:

This is called a "biphasic pulse train." Using your special cable, connect your iPhone or computer to your cockroach leg using the sound-out headphone jack.


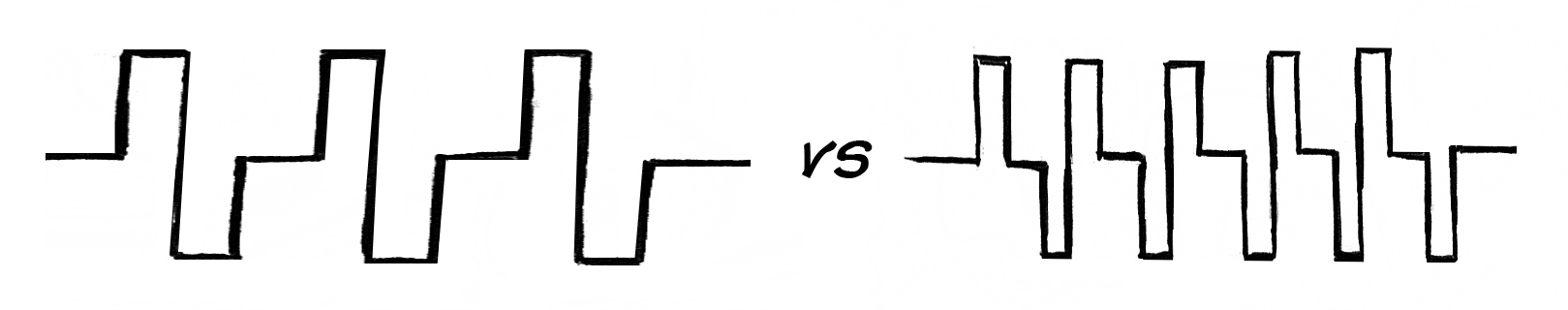
Putting your settings on “square wave,” adjust the rate (frequency)


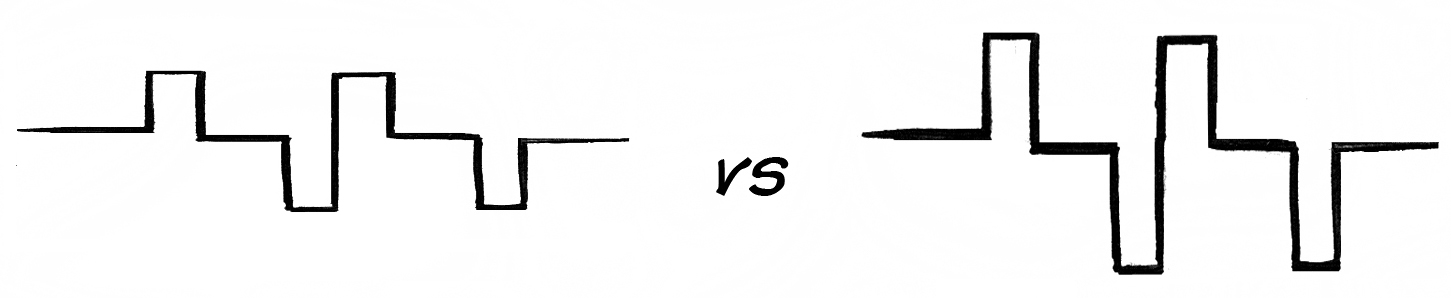
& volume (amplitude).

Can you find a “sweet spot” of the lowest volume & best frequency to cause evoked movement? Use the table below as a guide.


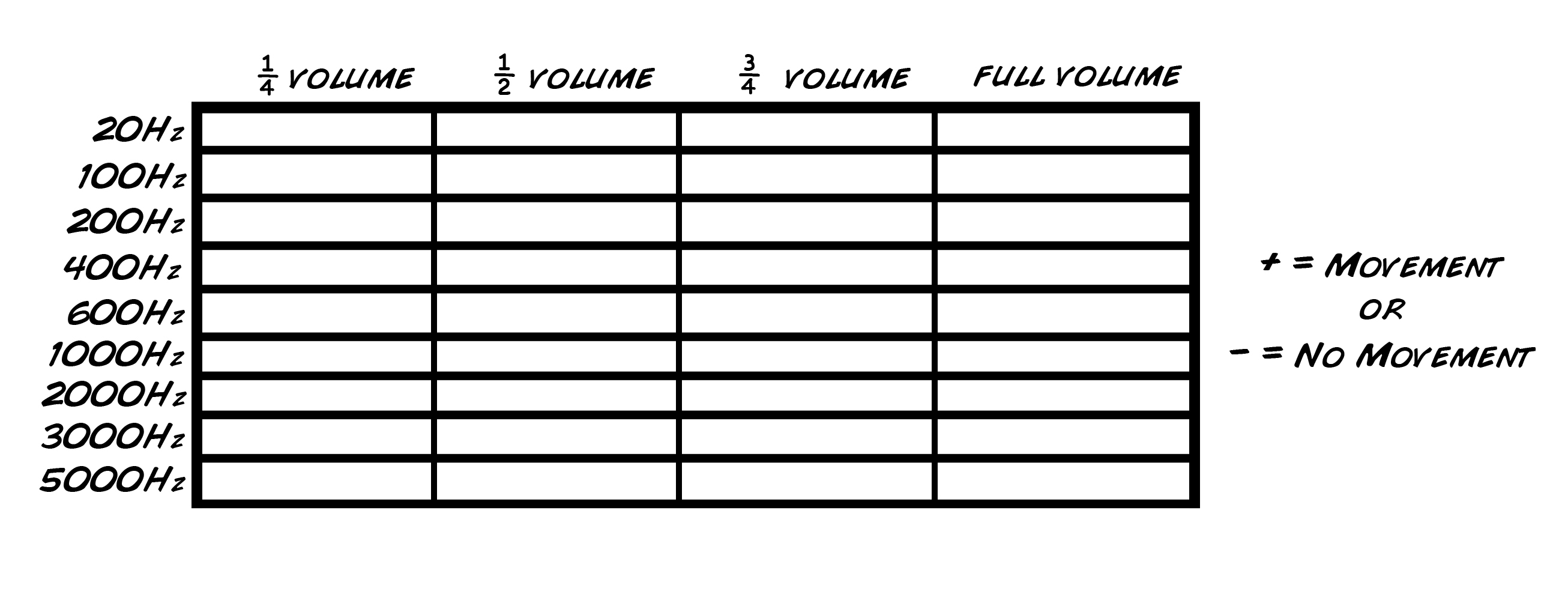

Supplement: File S4 — Student Hand-Out for Experiment III - How does your brain tell your muscles to move? (DOCX) [file pone.0030837.s005.docx]
