# Supplementary material for: The SpikerBox: A Low Cost, Open-Source BioAmplifier for Increasing Public Participation in Neuroscience Inquiry
Source: PLoS One. 2012 Mar 21;7(3):e30837. doi: 10.1371/journal.pone.0030837 (PMC3310049; doi:10.1371/journal.pone.0030837)
Supplement: File S5 — Student Hand-Out for Experiment IV - How do drugs affect neurons? (DOCX) [file pone.0030837.s006.docx]

**Experiment IV**

**How do drugs affect neurons?**

**Background:**


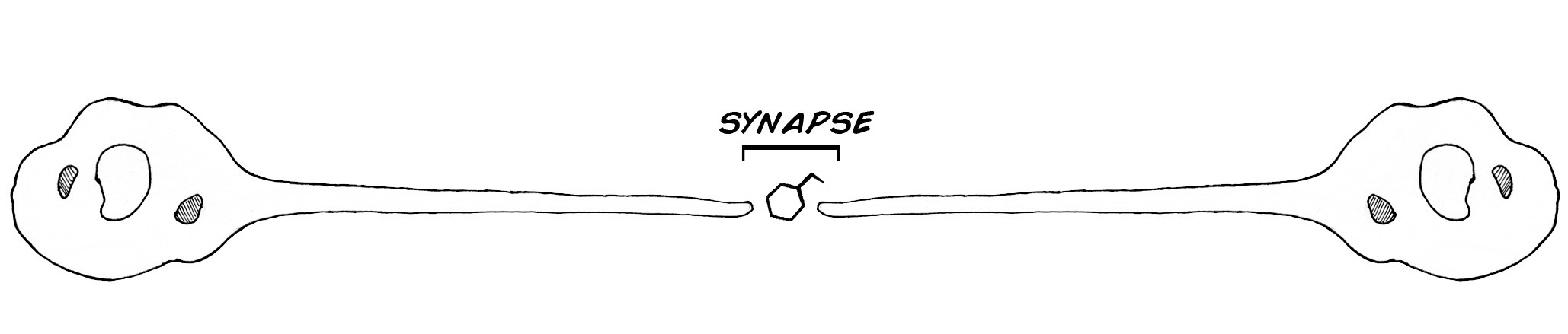
We have been studying “spikes” in the last few experiments, and we will continue to do so here, but now we are also going to study the synapse! Recall experiment I, where we explained that neurons communicate with a combination of electricity and chemicals. Consider two neurons:

Once a spike reaches the end the first neuron, it can cause the neuron to release “neurotransmitters” across the small distance between the two neurons called the “synapse.”  These neurotransmitters bind to receptors on the second neuron, which then cause the second neuron to begin firing spikes (or stop firing spikes, but let’s keep it simple for now). These receptors are very sensitive to electrical activity and certain chemicals. In fact, the very sensitivity of these receptors is how neurons, and ultimately you, learn!

In this experiment we will test the effect of neuroactive compounds on synapses. Obtaining drugs that affect neurons can be quite difficult, as they are often very dangerous (like the Batrachotoxins of poison dart frogs or the tetrodotoxins of Fugu Puffer Fish, both of which block sodium channels) or are drugs of abuse (like cocaine, which allows dopamine to stay in synapses longer than normal). But, we have access to two types of drugs we can use on our insects.


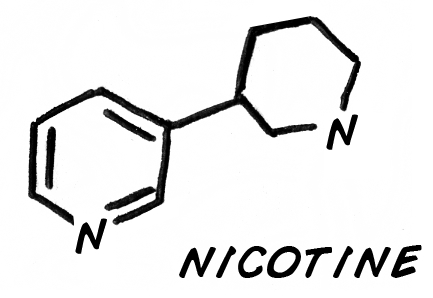
Nicotine and Monosodium glutamate!

Nicotine comes from the tobacco plant. Tobacco evolved nicotine to prevent insects from eating its leaves. Nicotine is a powerful acetylcholine receptor agonist; it amplifies the effect of acetylcholine binding to its receptors in synapses, causing a neuron to fire more (due to increased sodium ion influx).


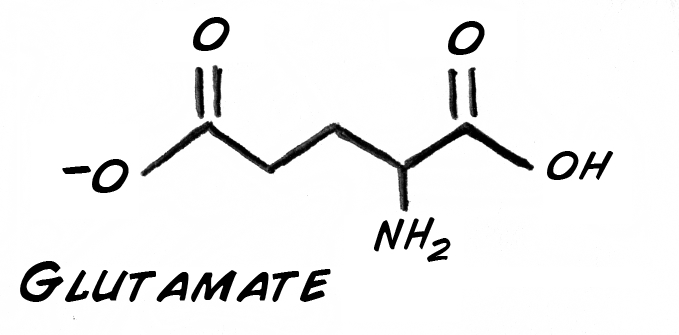
Whereas nicotine is a drug that acts on receptors that neurotransmitters bind to, Monosodium glutamate itself is a neurotransmitter. Once dissolved in water, it turns into positively charged sodium ions and negatively charged glutamate ions. Glutamate is normally part of the metabolic pathway of glycolysis (breakdown of sugar) and is readily available from the foods you eat.

In fact, over 80% of the synapses in your brain use glutamate as the excitatory neurotransmitter. In insects, is it excitatory as well? Let's find out!

**Procedure:**

To create your nicotine solution, take a cigarette or small cigar, remove all the shredded tobacco leaves, and place them in a small container (a clear pill bottle, for example). Fill the container with water, put the cap on, shake up the mixture, and allow it to sit for a couple days to extract the nicotine. Over time the solution should turn yellowish-brown. If you are in high school ask your teacher or parent to help you prepare this solution.


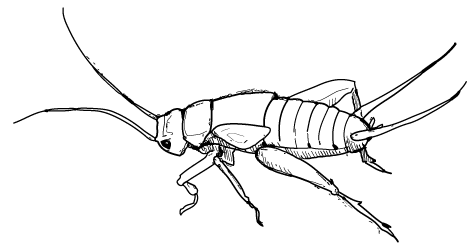
To create your glutamate solution, you need to find some monosodium glutamate! You can often find it at your friendly neighborhood Asian import grocery store. A pound should cost a couple dollars. Fill up a clear pill bottle about a quarter full of the MSG salt crystals, fill the remainder of the bottle with water, and shake thoroughly to dissolve the MSG. Note that not all of the MSG will dissolve, as you are making a saturated solution. Do some online research or ask your teacher to find out what a saturated solution is.

For reasons we haven't figured out (or really worked hard enough on yet), the cockroach leg preparation does not lend itself well to neuropharmacology experiments. Maybe you can prove us wrong, but for now, we are going to switch to a new species, the cricket cercal system!


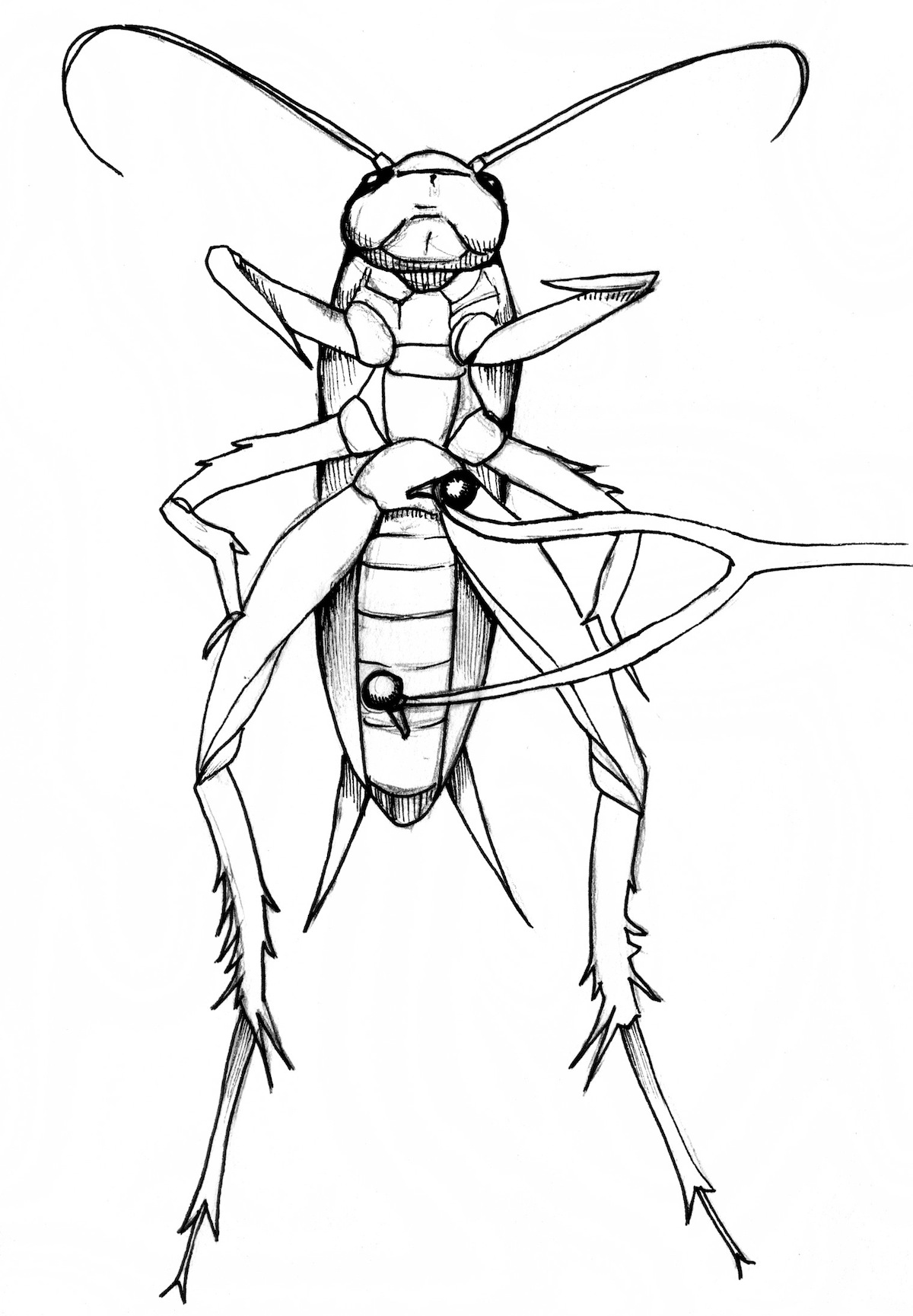
Crickets are readily available for local pet stores as feeder insects for lizards and frogs; they are a $1/dozen (we're not joking). Place your crickets on ice when you are ready to do an experiment. Take your two needles from your electrode and insert them along the central axis of the insect.

Now blow gently on the rear of the insect. You should see the cerci move from the pressure of your air puff. The cerci are sensing organs on the rear of the cricket that are sensitive to wind vibration. You should also hear an increase in the spiking activity on your SpikerBox. Note that these spikes are not as loud as the spikes you are used to hearing with the cockroach leg prep, but if you are lucky you should still hear them.

Now take a small syringe (you can also buy this at a local pharmacy over-the-counter) and inject a tiny bit of each solution into the cricket near one of the electrodes. What do you notice with the two different solutions? Can you explain any peculiar effects you hear with regards to spiking rate?

*This experiment is dedicated to Anuradha Rao, a neuroscientist who studied pharmacology and enjoyed educational outreach. Her memorial fund generously allowed Backyard Brains to present experiments and prototypes at the 2010 Society for Neuroscience Conference in San Diego, CA.*
